# Context Modulates Attention to Faces in Dynamic Social Scenes in Children and Adults with Autism Spectrum Disorder

**DOI:** 10.1007/s10803-021-05279-z

**Published:** 2021-10-08

**Authors:** Dzmitry A. Kaliukhovich, Nikolay V. Manyakov, Abigail Bangerter, Gahan Pandina

**Affiliations:** 1 Janssen Pharmaceutica NV, Turnhoutseweg 30, 2340 Beerse, Belgium; 2 Janssen Research & Development, LLC, Titusville, NJ USA

**Keywords:** Autism spectrum disorder, Biomarkers, Eye-tracking, Faces, Social attention

## Abstract

Individuals with autism spectrum disorder (ASD) have been found to view social scenes differently compared to typically developing (TD) peers, but results can vary depending on context and age. We used eye-tracking in children and adults (age 6–63) to assess allocation of visual attention in a dynamic social orientation paradigm previously used only in younger children. The ASD group (n = 94) looked less at the actor’s face compared to TD (n = 38) when they were engaged in activity (mean percentage of looking time, ASD = 30.7% vs TD = 34.9%; Cohen’s d = 0.56; *p* value < 0.03) or looking at a moving toy (24.5% vs 33.2%; d = 0.65; *p* value < 0.001). Findings indicate that there are qualitative differences in allocation of visual attention to social stimuli across ages in ASD.

ClinicalTrials.gov identifier: NCT02668991.

**Supplementary Information:**

The online version contains supplementary material available at 10.1007/s10803-021-05279-z.

## Introduction

Attention to faces and eyes gives insight into the emotional and mental states of others (Baron-Cohen et al., 2001; Peterson & Eckstein, 2012). Following cues such as gaze direction can help determine the focus and intentions of another person and infer meaning and context (Frischen et al., 2007; Senju et al., 2008). Modulation of attention to socially relevant stimuli is an essential element for engaging in successful social interaction (Freeth & Bugembe, 2019).

A core diagnostic feature of autism spectrum disorder (ASD) is impaired social interaction. There is extensive evidence that individuals with ASD show decreased attention to socially informative elements of visual scenes (e.g., people, faces, and gestures) compared with typically developing (TD) peers (Chita-Tegmark, 2016; Dawson et al., 1998; Frazier et al., 2017; Guillon et al., 2014; Klin et al., 2002). The cause for this decreased attention is likely multifactorial, particularly given the heterogeneity of clinical presentation of ASD. Researchers have postulated a number of contributing factors, ranging from biases in social perception, issues of motivation or salience (i.e., interest or preference), to perceptual or cognitive biases (Del Bianco et al., 2018; Hessels et al., 2018).

Eye-tracking studies are typically used to examine differences in gaze patterns between individuals with ASD and their neurotypical peers, or other developmental and psychiatric disorders. The main focus of these studies has been to assist with early detection and to understand early development. There are some indications that limited social attention may be predictive of later diagnosis and symptom severity in ASD (Campbell et al., 2014; Chawarska et al., 2013; Jones & Klin, 2013). A large meta-analysis of gaze differences between ASD and control participants in eye-tracking studies found that the overall effect sizes remained consistent across ages, indicating that social attention differences develop early and remain consistent throughout adolescence and adulthood (Frazier et al., 2017). However, studies vary widely in rigor, nature and complexity of visual stimuli. The meta-analysis revealed that methodological issues [for example, region-of-interest (ROI) definition, and differences in the type of stimuli used, such as complexity of social interaction] influenced effect size of the apparent deficit, regardless of age (Frazier et al., 2017). Other studies have indicated that results may be influenced by contextual factors and stimulus design, and that, in some contexts, individuals with ASD may have similar viewing patterns to those without ASD (Chevallier et al., 2015; Guillon et al., 2014; Hanley et al., 2015; Kwon et al., 2019). A recent comparative study looked specifically at developmental changes in attention to social interaction in a large sample of ASD and TD individuals across a range of paradigms that had previously demonstrated between group differences in social attention (Fujioka et al., 2020). They found that whole group differences in viewing patterns were not replicated in all paradigms. Specifically, the static faces with moving or non-moving mouth showed no diagnostic group differences in attention to eyes, but did show an interaction between group and age, indicating that there are qualitative differences in allocation of social attention across ASD development (Fujioka et al., 2020).

There is an increasing interest in the use of eye-tracking measures of social attention as potential biomarkers in clinical trials, as indicators of the presence or/and severity of ASD symptoms (Murias et al., 2018). In order for eye-tracking measures of social attention to be useful as biomarkers for stratification, diagnosis or change in symptom severity, they must be reliable and interpretable across the lifespan of ASD. One way to test social attention for its potential as a valid biomarker of ASD is to develop and use the same paradigm consistently within different age groups. For example, several research groups report on a set of dynamic stimuli where context was manipulated to include four experimental conditions comprising activity without speech, viewer-directed speech, joint attention, and moving non-social object (a toy), against a background of non-social potential distractor stimuli (Campbell et al., 2014; Chawarska et al., 2012, 2013; Wang et al., 2018). Use of different experimental conditions enabled investigation of contextual factors that modulate differences in allocation of attention within the groups of toddlers with or without ASD. Infants aged 6 months who were later diagnosed with ASD showed limited spontaneous attention to social scenes, particularly the actress’ body and the actress’ face, compared to other infants who did not receive the diagnosis later. For infants, the findings were across all contexts or experimental conditions (Chawarska et al., 2013). In contrast, in toddlers with ASD, in experimental conditions without eye contact and speech, attention distribution was similar to that of TD and developmentally delayed (DD) control participants. Only the dyadic bid condition, where the actor engaged in direct speech and eye contact, resulted in differences between the diagnostic groups. Toddlers with ASD showed less time looking at this scene overall, and specifically less time looking at faces and mouths compared to TD and DD controls (Chawarska et al., 2012). Using the same data and a different analytic approach (High-Cohesion Time Frames) to quantify atypical gaze, the ASD group gaze was found to be the least typical during the dyadic bid condition, and further that the atypicality was associated with more severe ASD symptoms (Wang et al., 2018). Similarly, in relation to symptom severity, increased attention to eyes and mouths at 2 years of age was shown to be positively related to functional outcome in the ASD group 1 year later (Campbell et al., 2014).

In this study, we employed an alternative version of the Chawarska’s paradigm (Plesa Skwerer et al., 2019) for the first time in older children and adults with ASD. The primary goal was to examine whether older children and adults with ASD differed from neurotypical peers in allocation of visual attention when viewing dynamic social videos. Specifically, we were interested in whether there was a potential modulation of those differences in gaze duration and quality by context. Based on previous findings in toddlers, we hypothesized that children and adults with ASD would spend less time looking at faces and face core features than the TD controls, with this difference being more prominent in the context with viewer-directed speech and eye contact. We also tested for relationships between the level of visual attention allocated to different experimental conditions or context and severity of ASD symptoms, as captured by several social behavior rating scales. Our hypothesis was that severity of ASD symptoms would correlate with time spent looking at faces.

## Methods

### Participants

Participants aged ≥ 6 years (range: 6–51) with a confirmed diagnosis of ASD based on clinical examination, caregiver interview and use of the Autism Diagnostic Observation Schedule, 2nd edition (ADOS-2) (Lord et al., 2012) were enrolled. Key exclusion criteria were a measured composite score on the Kaufmann Brief Intelligence Test-2 (KBIT-2) (Kaufman & Kaufman, 2004) of < 60, and history of or current significant medical illness. Each site also enrolled a control sample of TD participants, aged ≥ 6 years (range: 6–63), with a score in the normal range on the Social Communication Questionnaire (Rutter et al., 2003) who did not meet criteria for any major mental health disorder (American Psychiatric Association 2013), as assessed using the Mini-International Neuropsychiatric Interview (MINI) (Sheehan et al., 1998) for those 18 years old and above, and MINI-KID caregiver interview (Sheehan et al., 2010) for participants under 18 years. Age of 6 years was used as the cut off for this study, since it is the lower regulatory age bound for clinical studies in psychiatry. Note that the KBIT-2 data were collected only in individuals with ASD but not in TD controls. There were no exclusion criteria based specifically on vision. If participants required corrective lenses to view the screen and were able to obtain calibration on the measure at the start of each test set, then their eye-tracking data were included in the study.

Participants were enrolled within the framework of a large, observational, multi-center study that was conducted from 6 July 2015 to 14 October 2016 at nine study sites in the US (ClinicalTrials.gov identifier: NCT02668991). The study consisted of multiple free viewing tests (Bangerter et al., 2020a, b; Jagannatha et al., 2019; Manfredonia et al., 2019; Manyakov et al., 2018; Ness et al., 2017; Sargsyan et al., 2017) and both ASD and TD groups completed all of the tests during the same visit. In total, 136 individuals with ASD and 41 TD controls completed the study. Out of those, after exclusions due to technical or calibration failures, 94 (69.1%) individuals with ASD and 38 (92.7%) TD controls formed the study population. Table 1 lists participant characteristics. Further details on the participant characteristics can be found in Ness et al. (2017). Note that individuals with ASD that were included in analysis versus those excluded did not differ in the overall severity of ASD symptoms (Supplementary Table 11).

**Table 1:** Participant characteristics

Characteristic	ASD	TD
Sex, n (%)	94	38
Male	68 (72.3)	25 (65.8)
Female	26 (27.7)	13 (34.2)
* χ*^*2*^ test*, p* value	0.16	
Age, years		
Mean (SD)	14.5 (7.3)	16.7 (13.6)
Median (range)	12.0 (6–51)	12.0 (6–63)
Kolmogorov–Smirnov test, *p* value	0.69	
ADOS-2 Total score, mean (SD, range)	7.5 (1.7, 4–10)	–
KBIT-2 IQ composite score, mean (SD, range)	100.3 (19.2, 60–136)	–

The reported *p* values are two-sided. ‘n’ indicates the number of participants

*ADOS-2* Autism Diagnostic Observation Schedule 2nd edition, *ASD* autism spectrum disorder, *IQ* intelligence quotient, *KBIT-2* Kaufman Brief Intelligence test-2, *SD* standard deviation, *TD* typically developing

### Stimuli

**Fig. 1:**
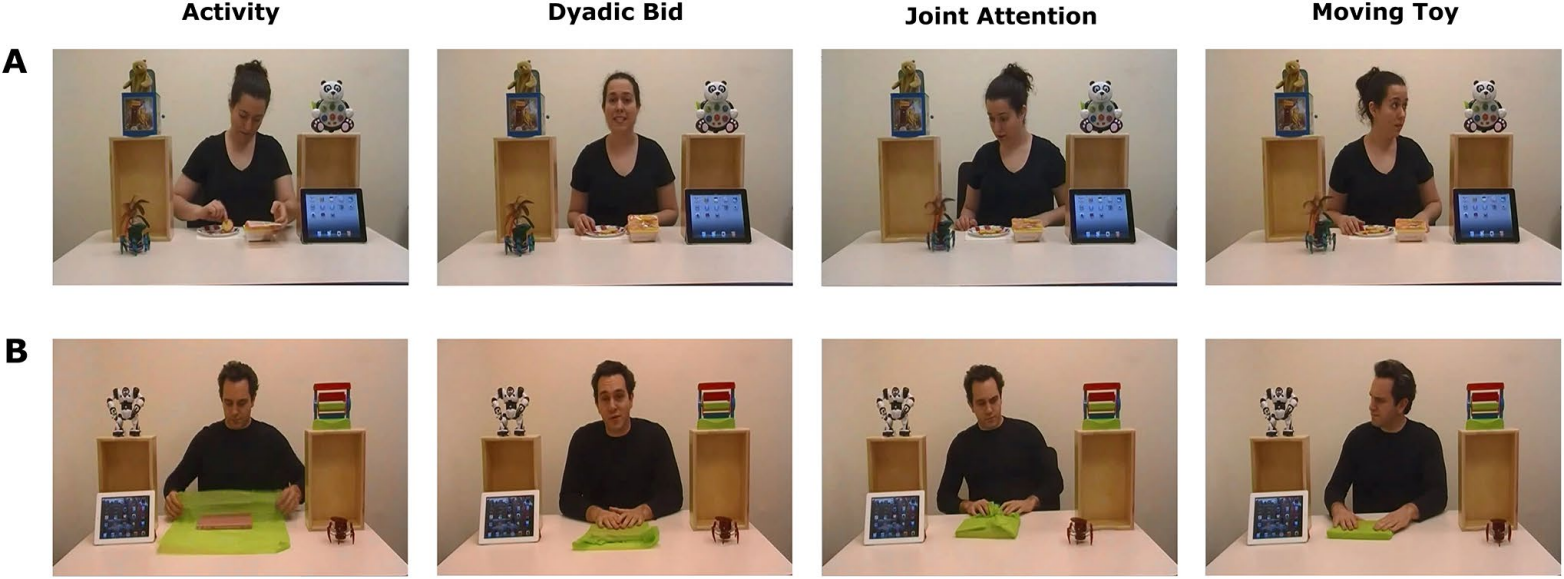
Stimuli and stimulus conditions. Each of the four stimulus conditions is represented by a single frame from each of the two video stimuli. One video shows a female actor (**A**), whereas the other shows a male actor (**B**). Stimulus conditions are indicated in bold above the frames in the first row (**A**)

The stimuli were adopted from the alternative version of the Chawarska’s paradigm (Plesa Skwerer et al., 2019) and were similar to those first reported (Chawarska et al., 2012). Each participant viewed two videos, with one video presenting a female actor (total duration 73 s) and another one presenting a male actor (90 s). The presentation order of the two stimuli was random across participants. In each video, an actor was filmed while sitting in front of a table in a brightly lit room with barren walls (Fig. 1). There was a tablet computer standing on the table on one side of the actor and a multi-legged walking robot toy on the other. The table also accommodated two wooden stands, with one on each side of the actor. A single toy was placed on top of each of these stands. All toys, except for the walking robot toy, and the stands remained static throughout the videos. The position of the walking robot toy alternated across the videos.

The stimuli were designed to depict an actor performing an activity, occasionally looking at the camera and trying to engage a viewer or looking at the toys positioned in the four corners of the screen, with one toy either remaining still or moving. Specifically, each video consisted of four stimulus conditions (Fig. 1), with each condition being presented over multiple episodes. Each stimulus condition represented an instance of the behavior associated with that condition. In the *Activity* condition, an actor looked down at the table while either making a snack (female actor, five episodes, total duration 45 s) or wrapping a book in paper (male actor, 4 episodes, total duration 63 s). No direct actor’s gaze or speech cues were present in this condition. In the *Dyadic Bid* condition, an actor lifted up the head, looked toward the camera and engaged in a viewer-directed speech, thus resembling a bid for dyadic attention (female vs. male actor: 3 episodes in each video, 14 vs. 16 s). The content of actor’s speech was related to the events presented in a video and included a greeting to the viewer (e.g., “Oh, hello!”), comments on the performed activity (“It’s my friend’s birthday today. I bought him a book.”) and its completion (“It’s done now. I think I like it.”). In the *Joint Attention* condition, the walking robot toy suddenly began to move on the table while making noise generated by its actuators. This immediately interrupted actor’s activity and made an actor turn toward the toy. An actor was continuously maintaining attention on the moving robot toy until it returned to the initial position and stopped. After the toy stopped, an actor continued to carry out his/her activity (female vs. male actor: 1 episode in each video, 9 vs. 6 s). The *Moving Toy* condition was the same as the *Joint Attention* condition, except that an actor turned toward and was continuously looking at the toy placed on the wooden stand on the diametrically opposite side to the walking robot toy (one 5-s long episode in each video). The walking robot toy moved only in the experimental conditions of *Joint Attention* and *Moving Toy* and not in the other two.

Episodes associated with different stimulus conditions were interleaved throughout the videos in order to provide variation and increase participant’s interest. Supplementary Table 1 lists the order of episodes of different stimulus conditions in each video as well as their duration. To avoid extraneous attentional cues and disruption in processing of social scenes (Hirose et al., 2010), each video consisted of a stream of activities with no breaks to re-engage or re-center participant’s visual attention. This type of display required participants to adjust their viewing patterns depending on the context, as they would do in real-life circumstances, without the benefit of extraneous cues directing their attention to the screen in general or to any of the specific elements of the scene.

### Procedure

Participants sat in a comfortable chair approximately 60 cm from a 23-inch computer screen (1920 × 1080 pixels). The height of the chair and screen were adjusted to ensure that participants’ eyes were level with the center of the screen. Eye-tracking data were collected using the Tobii X2 eye tracker, with a sampling rate of 30 Hz, mounted below the screen. iMotions Biometric Research Platform (https://imotions.com/) was used for stimuli presentation, data synchronization, and automatic calibration. Participants could freely observe presented stimuli. To ensure high accuracy of the eye movement recordings (Blignaut & Wium, 2014), each recordings session was preceded by a five-point calibration procedure that consisted of presentations of animated cartoon characters paired with an auditory cue. The calibration procedure was aimed to reach the mean distance between the participant’s gaze direction and the target points of less than 0.5° of visual angle.

### Behavior Rating Scales

Scale data were collected concurrently with eye-tracking data. Parents or caregivers of individuals with ASD were required to spend at least 3 days per week with participants, to have sufficient opportunity (over several days) to observe recent behavior and symptoms and allow accurate reporting. They completed the following scales:

- Autism Behavior Inventory (ABI) assesses changes in core and associated ASD symptoms (Bangerter et al., 2017).
- Aberrant Behavior Checklist—Community (ABC) assesses general behaviors (Aman & Singh, 2017; Aman et al., 2004)
- Child Adolescent Symptom Inventory (CASI) assesses a range of behaviors related to common emotional and behavior disorders (Gadow & Sprafkin, 1997).
- Social Responsiveness Scale 2™ (SRS-2) identifies presence and severity of social impairment due to ASD (Constantino et al., 2003).
- Repetitive Behavior Scale-Revised (RBS-R) provides a quantitative measure of the full spectrum of repetitive behaviors (Lam & Aman, 2007).

Each of the above scales and ADOS-2 consist of several subscales that reflect different ASD symptoms.

### Data Analysis

Standard ROI analysis techniques were adopted for the analysis of gaze patterns. The variables of interest were the proportions of time spent looking at each of the regions (i.e., the total amount of time the participant’s point of regard was located within each region) and included (a) overall attention to the scene (% *Valid Time*), (b) proportion of overall attention (*% looking time*) directed toward an actor (including *Body*, *Hands/Activity* area, *Face* as a whole and its core features *Eyes*, *Mouth*, and *Hair*), and (c) attention toward distractors (*Toys*) and background (*Background*). All proportions (*% looking time*) were computed for each stimulus condition separately. The proportion of total looking time (% *Valid Time*) in each of the four stimulus conditions was standardized by the total duration of the video display in that condition. The remaining variables were standardized by the total looking time at the scene per stimulus condition. All analyses were based on *% looking time* for different ROIs averaged across the two video stimuli.

A linear mixed-effects model was used to compare the level of overall visual attention across the four stimulus conditions between the two groups of participants. The model included % *Valid Time* as a dependent variable, and participant’s age and sex, participant group, stimulus condition and interaction between these two factors as fixed effects:

$$\% \,Valid\,time \sim Age + Sex + Group + Condition + Group \times Condition.$$

To account for within-participant variability in % *Valid Time*, the model included a participant identifier as the random intercept. The model was fit by using the R package “nlme” (Pinheiro et al., 2018) and maximizing the restricted log-likelihood function. Significance of the fixed effects was assessed using the analysis of variance type III sum of squares and the Wald χ^2^ test (Supplementary Table 2), as implemented in the R package “car” (Fox & Weisberg, 2011). Post hoc pair-wise comparisons were performed using the Tukey–Kramer correction for multiple comparisons (Supplementary Table 3). The least-squares mean estimates and their 95% two-sided confidence intervals for different levels of the modelled categorical factors were obtained with the R package “lsmeans” (Lenth, 2016) (Supplementary Table 4). This package was also used to run post hoc pair-wise comparisons.

A similar approach was applied to compare percentage of time spent looking at different ROIs between the two groups of participants. The data of each of the four stimulus conditions were modelled separately. Each linear mixed-effects model included percentage looking time as a dependent variable, and participant’s age and sex, participant group, ROI and an interaction between these two factors as fixed effects:

$$\% \,Looking\,time \sim Age + Sex + Group + ROI + Group \times ROI.$$

Each model included a participant identifier as the random intercept. Tests of significance of the fixed effects and post hoc pair-wise comparisons were performed in the same manner as in the model of overall visual attention (see above). Two separate sets of ROIs were tested. The first set comprised *Background*, *Body*, *Hands/Activity*, *Face* and *Toys* that constituted the entire visual scene (Supplementary Tables 5 and 6), whereas the second one represented the core features of faces and included *Eyes*, *Mouth* and *Hair* (Supplementary Tables 7 and 8). Note that qualitatively similar results were obtained when incorporating % *Valid Time* per stimulus condition as an additional factor into the models (data not shown). Given a higher complexity of these models and similarity of the results they produced, all results reported in this article were obtained with the models described above that did not include % *Valid Time*. Since no linear mixed-effects model revealed a significant effect of either participant’s age or sex on *% looking time* (Supplementary Tables 5 and 7), Cohen’s d was computed to assess the effect of participant group on *% looking time* for each ROI and stimulus condition separately (Tables 2 and 3).

**Table 2:** Percentage of time spent looking at regions of interest for each stimulus condition and group of participants separately

Condition	Group	Region-of-interest				
		Background	Body	Hands/activity	Face	Toys
Activity	ASD	3.7 (2.9)	3.9 (2.0)	47.9 (10.2)	30.7 (8.2)	13.7 (6.8)
	TD	3.0 (1.9)	3.6 (1.5)	46.3 (7.1)	34.9 (5.1)	12.2 (7.6)
	Cohen’s d	0.27	0.17	0.17	0.56	0.22
	*p* value	1.00	1.00	0.95	**0.03**	0.96
Dyadic bid	ASD	4.0 (4.8)	4.6 (4.9)	33.8 (14.9)	49.3 (15.9)	8.4 (7.5)
	TD	2.7 (3.7)	3.9 (2.5)	39.4 (12.3)	48.6 (12.0)	5.4 (5.8)
	Cohen’s d	0.28	0.16	0.40	0.05	0.42
	*p* value	1.00	1.00	0.12	1.00	0.89
Joint attention	ASD	5.4 (5.4)	3.2 (3.1)	36.5 (12.1)	24.5 (13.0)	30.4 (14.9)
	TD	4.7 (5.5)	3.1 (2.5)	34.6 (8.7)	33.2 (13.8)	24.3 (13.3)
	Cohen’s d	0.12	0.03	0.17	0.65	0.42
	*p* value	1.00	1.00	1.00	**10**^**–3**^	0.09
Moving toy	ASD	3.4 (4.3)	2.7 (2.8)	12.9 (11.3)	25.5 (16.6)	55.4 (19.7)
	TD	2.0 (2.9)	3.1 (3.0)	14.0 (8.8)	26.6 (15.8)	54.2 (17.4)
	Cohen’s d	0.35	0.13	0.10	0.07	0.06
	*p* value	1.00	1.00	1.00	1.00	1.00

Cells contain mean % looking time, with the corresponding standard deviations being provided in parentheses

*p* values correspond to between-group comparisons of % looking time and are obtained using linear mixed-effects models and the Tukey–Kramer correction for multiple comparisons. *p* values below 0.05 are highlighted in bold

*ASD* autism spectrum disorder, *TD* typically developing

**Table 3:** Percentage of time spent looking at the core features of faces for each stimulus condition and group of participants separately

Condition	Group	Region-of-interest		
		Eyes	Mouth	Hair
Activity	ASD	4.9 (4.8)	24.7 (7.5)	1.1 (1.8)
	TD	5.7 (4.3)	28.5 (5.8)	0.8 (1.0)
	Cohen’s d	0.17	0.53	0.21
	*p* value	0.95	**2 × 10**^**–3**^	1.00
Dyadic bid	ASD	24.8 (17.2)	19.5 (10.0)	3.5 (7.3)
	TD	25.4 (18.0)	21.0 (12.0)	1.2 (2.7)
	Cohen’s d	0.03	0.14	0.32
	*p* value	0.99	0.99	0.92
Joint attention	ASD	11.5 (10.4)	7.8 (7.3)	5.2 (7.7)
	TD	14.9 (13.8)	11.9 (7.7)	6.3 (8.2)
	Cohen’s d	0.30	0.56	0.14
	*p* value	0.41	0.20	0.99
Moving toy	ASD	10.5 (9.1)	11.4 (11.6)	3.6 (7.5)
	TD	12.3 (10.4)	12.8 (11.5)	1.6 (3.4)
	Cohen’s d	0.19	0.12	0.31
	*p* value	0.93	0.98	0.86

Cells contain mean % looking time, with the corresponding standard deviations being provided in parentheses

*p* values correspond to between-group comparisons of % looking time and are obtained using linear mixed-effects models and the Tukey–Kramer correction for multiple comparisons. *p* values below 0.05 are highlighted in bold

*ASD* autism spectrum disorder, *TD* typically developing

All reported correlations (r_S_) between either % *Valid Time* or *% looking time* for different ROIs and the KBIT-2 IQ composite score were Spearman correlations (Supplementary Table 9). Relationships between % *Valid Time*, *% looking time* for different ROIs on one hand and severity of ASD symptoms were assessed using Spearman partial correlations, with participant’s age, sex and KBIT-2 IQ composite score serving as covariates (Supplementary Table 10). Spearman partial correlations and corresponding *p* values were computed using the R package “ppcor” (Kim, 2015). The choice of Spearman correlations was attributed to a generally lower susceptibility of this type of correlations to potential outliers present in the data, as compared to Pearson correlations. All reported *p* values were two-sided. No correction for multiple comparisons was performed. The exploratory nature of this study prevented specification of all tests prior to their conduct. As a result, the exact cut-off for significant *p* values in each analysis is debatable. For this reason, *p* values were reported “as is” with values < 0.05 considered significant.

## Results

### Level of Overall Visual Attention Across Stimulus Conditions

The level of overall visual attention (% *Valid Time*) was high in each of the two groups of participants and significantly varied across stimulus conditions (*p* value < 0.0005) (Supplementary Table 2 and Fig. 1). Mean % *Valid Time* in individuals with ASD ranged from 85.3% in the condition *Activity* to 87.0% in the experimental conditions of *Joint Attention* and *Moving Toy* (Supplementary Table 4). Mean % *Valid Time* in TD controls varied between 87.9% in the condition *Activity* and 90.4% in the condition *Dyadic Bid*. Post hoc comparisons revealed no significant difference in % *Valid Time* between the two groups of participants in any of the four stimulus conditions (all *p* values > 0.59) (Supplementary Table 3).

### % Looking Time for ROIs that Constituted the Visual Scene

**Fig. 2:**
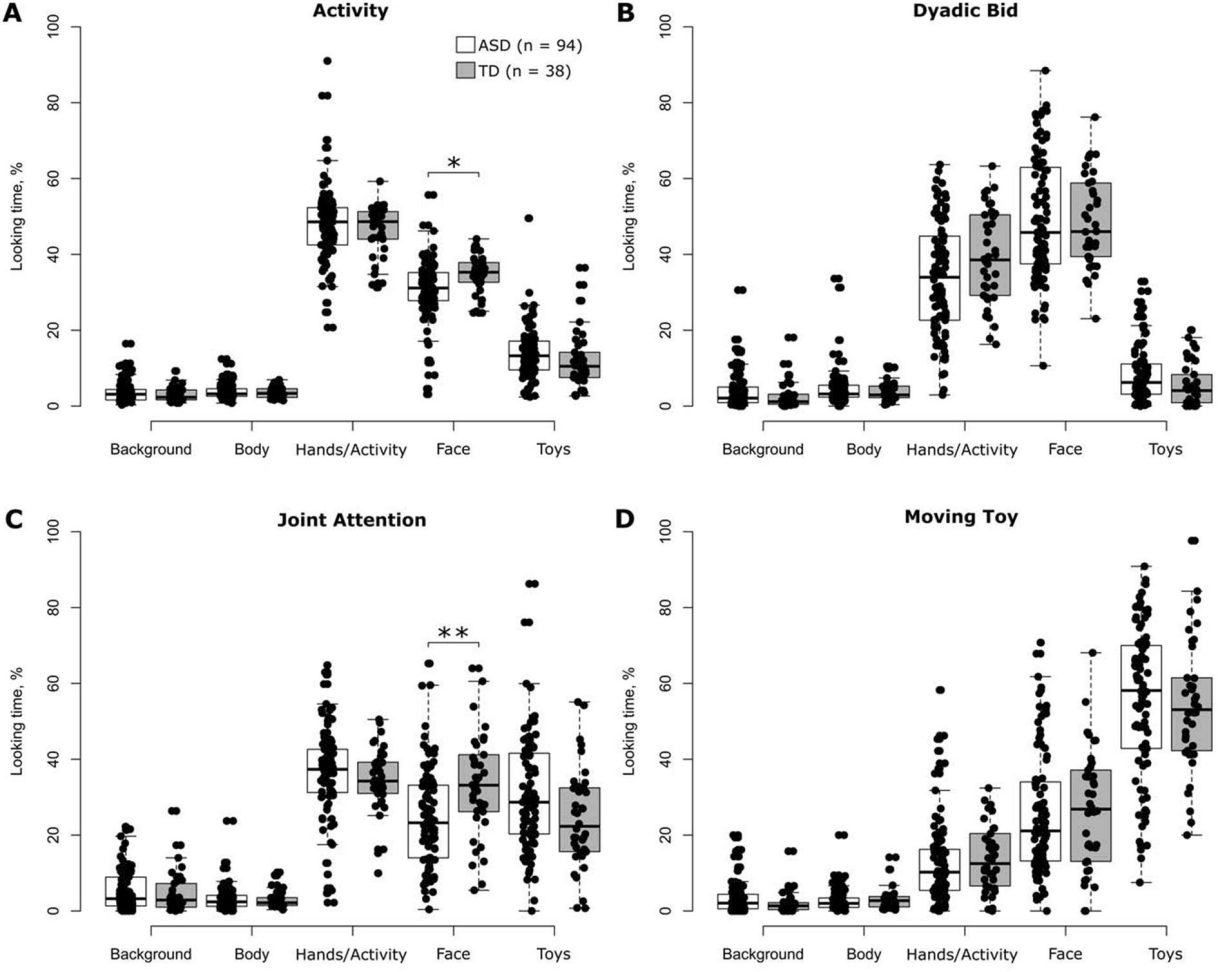
Distributions of *% looking time* for each stimulus condition, group of participants and region-of-interest separately. The data are summarized in a form of boxplots. Black dots in each panel denote individual participants. The white and grey boxplots correspond to the data of individuals with ASD and TD controls, respectively. ‘n’ indicates the number of participants. Only significant between-group differences are shown. *p value < 0.05, **p value < 0.005 (corrected for multiple comparisons for each stimulus condition separately using the Tukey–Kramer method) (Color figure online)

In each of the four stimulus conditions tested separately, *% looking time* significantly varied across ROIs (all *p* values < 10^−15^) (Supplementary Table 5), and this held true in each of the two groups of participants (Fig. 2). The participants spent least time looking at *Background* and *Body* across the four stimulus conditions (Table 2). In contrast, *% looking time* for the remaining three ROIs was considerably higher and spanned a broader range of values (Table 2), thus suggesting that changes in allocation of visual attention across experimental conditions were mainly subject to these ROIs. Participant’s age or sex did not significantly modulate *% looking time* for any of the stimulus conditions (all *p* values = 1.0) (Supplementary Table 5). Although participant group did not significantly modulate *% looking time* for different ROIs in any of the four stimulus condition models (all *p* values > 0.52), the interaction between participant group and ROI reached statistical significance in all experimental conditions (all *p* values < 0.02), except *Moving Toy* (*p* value = 0.90) (Supplementary Table 5). Post hoc comparisons of *% looking time* per ROI between the two groups of participants performed for each stimulus condition separately revealed a significantly lower *% looking time* for *Face* in individuals with ASD as compared to TD controls in the stimulus conditions *Activity* (mean *% looking time*, ASD vs. TD: 30.7% vs. 34.9%; Cohen’s d = 0.56; *p* value < 0.03) and *Joint Attention* (mean *% looking time*, ASD vs. TD: 24.5% vs. 33.2%; Cohen’s d = 0.65; *p* value < 0.01) (Table 2). No other ROI showed a significant difference in *% looking time* between the two groups of participants in any of the four stimulus conditions.

### % Looking Time for the Core Features of Faces

**Fig. 3:**
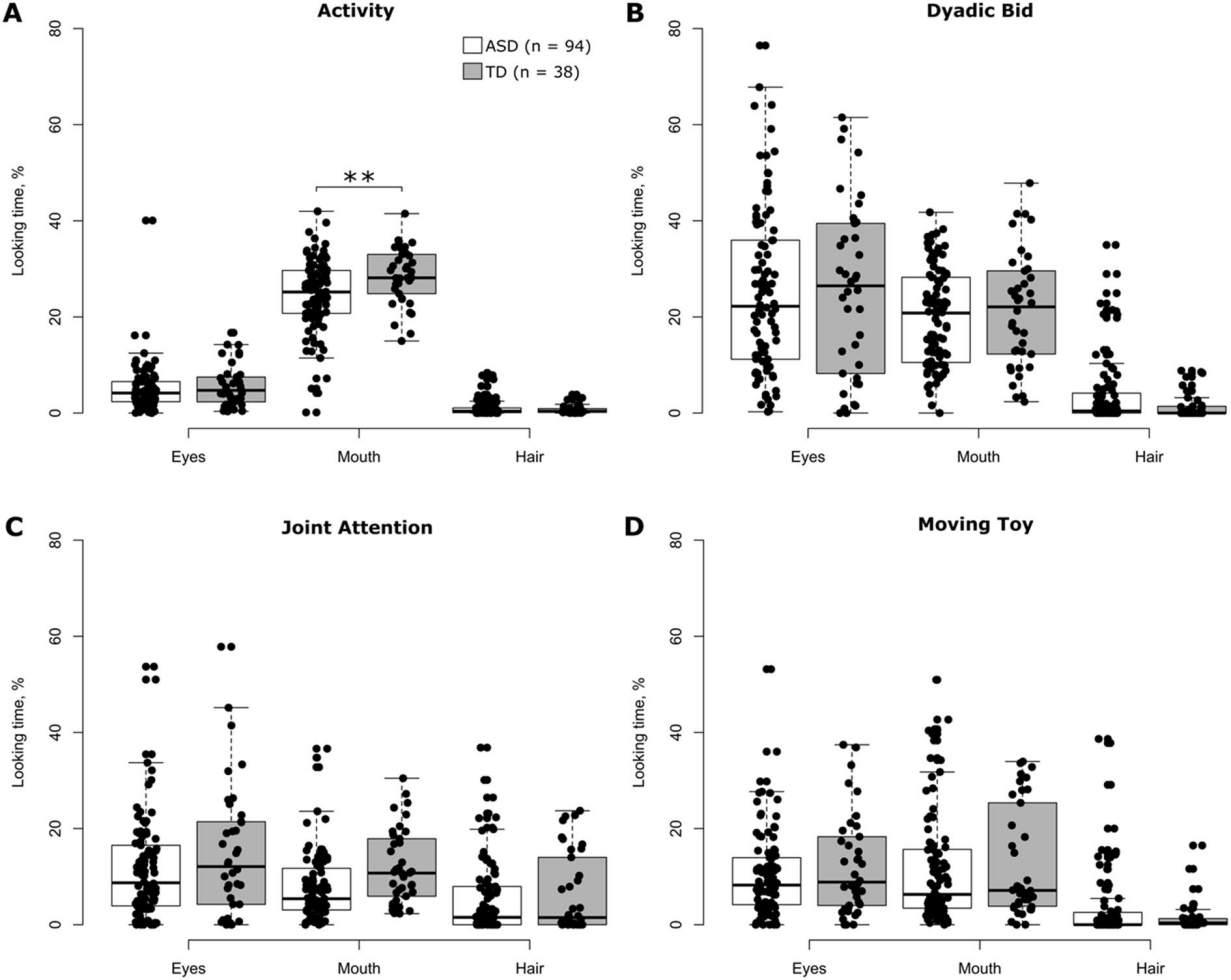
Distributions of *% looking time* for the core features of faces for each stimulus condition and group of participants separately. The data are summarized in a form of boxplots. Black dots in each panel denote individual participants. The white and grey boxplots correspond to the data of individuals with ASD and TD controls, respectively. ‘n’ indicates the number of participants. Only significant between-group differences are shown. **p value < 0.005 (corrected for multiple comparisons for each stimulus condition separately using the Tukey–Kramer method) (Color figure online)

Restricting analyses to the core features of faces (*Eyes*, *Mouth*, and *Hair)* showed a significant modulation of *% looking time* by ROI in each of the four stimulus conditions (all *p* values < 0.0001) (Supplementary Table 7), and this held true in each of the two groups of participants (Fig. 3). Both groups of participants spent least time looking at *Hair* (Table 3). This suggests that changes in allocation of visual attention to faces across conditions were mainly subject to *Eyes* and *Mouth*. Neither participant’s age (all *p* values > 0.28) nor sex (all *p* values > 0.13) significantly modulated *% looking time* for different ROIs in any of the four stimulus conditions (Supplementary Table 7). Similarly, participant group showed no effect on *% looking time* for different ROIs in any of the four stimulus conditions (all *p* values > 0.06) (Supplementary Table 7). The interaction between participant group and ROI reached statistical significance only in the condition *Activity* (*p* value < 0.01) and not in the other three (all *p* values > 0.26) (Supplementary Table 7). Post hoc comparisons of *% looking time* per ROI between the two groups of participants performed for each stimulus condition separately revealed a significantly lower *% looking time* for *Mouth* in individuals with ASD as compared to TD controls in the stimulus condition *Activity* (mean *% looking time*, ASD vs. TD: 24.7% vs. 28.5%; Cohen’s d = 0.53; *p* value < 0.002) (Table 3). Mean *% looking time* for *Eyes* and *Mouth* was consistently lower in individuals with ASD than in TD controls across the four stimulus conditions. Moreover, the same ranking of mean *% looking time* was observed across the four stimulus conditions in both groups of participants for *Eyes* (in ascending order: *Activity*, *Moving Toy*, *Joint Attention*, *Dyadic Bid*) and *Mouth* (*Joint Attention*, *Moving Toy*, *Dyadic Bid*, *Activity*) (Table 3). Similar results were obtained when using median *% looking time* (data not shown). Altogether this suggests a similar pattern of visual attention allocation on these two core features of faces in both groups of participants, with the level of attention being lower in individuals with ASD as compared to TD controls.

### Relationship Between Participant’s IQ and % Looking Time for Different ROIs

The data on participants’ intelligence, as assessed by KBIT-2, were collected only in individuals with ASD. This prevented the use of these data in comparisons of % *Valid Time* and *% looking time* between the two groups of participants. When relating % *Valid Time* and *% looking time* for different ROIs to the KBIT-2 IQ composite score in the ASD sample for each of the four stimulus conditions separately (n = 39 correlation coefficients computed), no significant relationship was observed (all *p* values > 0.10) (Supplementary Table 9). Repeating the same analysis in the groups of individuals with ASD with a specific level of IQ (n = 3 levels tested) revealed two significant relationships out of 117 tested ones (Supplementary Table 9). The KBIT-2 IQ composite score positively correlated (1) with % *Valid Time* in the condition *Dyadic Bid* (r_S_ = 0.280, *p* value < 0.04) in the group of individuals with ASD with average IQ (range: 85–115; n = 54 participants) and (2) with *% looking time* for *Toys* in the condition *Moving Toy* (r_S_ = 0.482, *p* value < 0.03) in the group of individuals with ASD with low IQ (range: 60–84; n = 21 participants) (Supplementary Fig. 2). Note, however, that these correlations would not remain significant if correction for multiple testing was performed, thus ruling out the explanation of significant differences observed in *% looking time* by participant’s IQ (Figs. 2 and 3).

### Relationship Between Severity of ASD Symptoms and % Looking Time for Different ROIs

To test whether overall severity of ASD symptoms affected time spent (%) looking at the presented stimuli or at a specific ROI, % *Valid Time* and *% looking time* for different ROIs were assessed for correlation with the total score of multiple behavior rating scales (n = 5). The correlation coefficients were computed for each ROI, stimulus condition and behavior rating scale separately. Out of 120 computed correlation coefficients (Table 4), only 11 (9.2%) reached statistical significance (all *p* values < 0.05). Greater overall severity of ASD, as assessed using the ABI scale, was associated with lower overall visual attention (% *Valid Time*) to the presented stimuli (stimulus condition *Joint Attention*: r_S_ =  − 0.250, *p* value < 0.02; *Moving Toy*: r_S_ =  − 0.225, *p* value < 0.03). Furthermore, individuals with greater severity of ASD symptoms, as assessed using the ADOS-2 scale, tended to look more at *Background* (r_S_ = 0.227, *p* value < 0.03) and *Toys* (r_S_ = 0.288, *p* value < 0.01) in the condition resembling a bid for dyadic attention (*Dyadic Bid*). Greater overall severity of ASD symptoms was associated with an attenuated interest in *Toys* when the actors in the presented video stimuli allocated their attention on the walking robot toy (stimulus condition *Joint Attention*). This was evident on both ABI (r_S_ =  − 0.261, *p* value < 0.01) and SRS-2 scales (r_S_ =  − 0.283, *p* value < 0.01). In addition, overall severity of ASD symptoms in the condition *Joint Attention* significantly correlated with *% looking time* for *Face* (RBS-R: r_S_ = 0.216, *p* value < 0.04) and *Hands/Activity* (ADOS-2: r_S_ =  − 0.218, *p* value < 0.04). After the actors diverted their attention away from the walking robot toy (stimulus condition *Moving Toy*), more severely affected individuals with ASD tended to pay more attention to *Toys*, as was evident on the ADOS-2 scale (r_S_ = 0.246, *p* value < 0.02). Moreover, overall severity of ASD symptoms in the condition *Moving Toy* was also significantly associated with *% looking time* for *Background* (ABI: r_S_ = 0.265, *p* value < 0.01) and *Face* (ADOS-2: r_S_ =  − 0.222, *p* value < 0.03). No significant relationship between *% looking time* for *Body* and overall severity of ASD symptoms was observed (all *p* values > 0.09) for any of the four stimulus conditions and five behavior rating scales. Supplementary Table 10 lists correlations between *% looking time* for different ROIs and individual ASD symptoms. Only 37 of 696 computed correlation coefficients (5.3%) were significant.

**Table 4:** Correlations between % *Valid Time*, *% looking time* for different regions of interest and the total score of behavior rating scales in individuals with ASD

Condition	Region-of-interest					
	% Valid time	Background	Body	Hands/activity	Face	Toys
Autism behavior inventory (n = 93)						
Activity	− 0.205 (0.05)	0.147 (0.17)	0.179 (0.09)	− 0.081 (0.45)	0.067 (0.53)	− 0.081 (0.45)
Dyadic bid	− 0.146 (0.17)	0.117 (0.27)	0.014 (0.89)	0.013 (0.90)	− 0.056 (0.60)	− 0.016 (0.88)
Joint attention	**− 0.250 (0.02)**	0.158 (0.14)	0.080 (0.45)	− 0.023 (0.83)	0.190 (0.07)	**− 0.261 (0.01)**
Moving toy	**− 0.225 (0.03)**	**0.265 (0.01)**	− 0.020 (0.85)	0.027 (0.80)	0.054 (0.61)	− 0.148 (0.16)
Autism diagnostic observation schedule, 2nd edition (n = 94)						
Activity	− 0.076 (0.47)	0.090 (0.40)	0.006 (0.96)	− 0.122 (0.25)	− 0.133 (0.21)	0.193 (0.07)
Dyadic bid	− 0.086 (0.42)	**0.227 (0.03)**	− 0.018 (0.87)	− 0.173 (0.10)	− 0.001 (0.99)	**0.288 (0.01)**
Joint attention	− 0.081 (0.45)	− 0.002 (0.98)	− 0.002 (0.98)	**− 0.218 (0.04)**	− 0.047 (0.66)	0.177 (0.09)
Moving toy	− 0.141 (0.18)	0.099 (0.35)	− 0.167 (0.11)	− 0.106 (0.32)	**− 0.222 (0.03)**	**0.246 (0.02)**
Child adolescent symptom inventory—anxiety (n = 94)						
Activity	− 0.050 (0.63)	− 0.029 (0.79)	0.045 (0.67)	− 0.102 (0.33)	0.170 (0.11)	− 0.004 (0.97)
Dyadic bid	0.074 (0.49)	0.025 (0.81)	− 0.100 (0.35)	0.114 (0.28)	− 0.071 (0.51)	− 0.067 (0.53)
Joint attention	− 0.037 (0.73)	− 0.106 (0.32)	0.112 (0.29)	0.063 (0.55)	0.106 (0.32)	− 0.140 (0.18)
Moving toy	− 0.078 (0.46)	0.160 (0.13)	0.059 (0.58)	0.153 (0.15)	− 0.082 (0.44)	− 0.015 (0.89)
Repetitive behavior scale—revised (n = 94)						
Activity	− 0.084 (0.43)	− 0.006 (0.95)	0.028 (0.79)	− 0.120 (0.26)	0.107 (0.32)	0.030 (0.78)
Dyadic bid	− 0.070 (0.51)	0.023 (0.83)	− 0.062 (0.56)	− 0.098 (0.36)	0.114 (0.28)	− 0.027 (0.80)
Joint attention	− 0.129 (0.22)	− 0.066 (0.53)	0.023 (0.83)	− 0.085 (0.42)	**0.216 (0.04)**	− 0.100 (0.35)
Moving toy	− 0.067 (0.53)	0.125 (0.24)	− 0.093 (0.38)	− 0.011 (0.92)	0.091 (0.39)	− 0.086 (0.42)
Social responsiveness scale 2 (n = 93)						
Activity	− 0.032 (0.76)	0.005 (0.96)	− 0.053 (0.62)	0.015 (0.89)	0.076 (0.47)	− 0.082 (0.44)
Dyadic bid	0.038 (0.72)	0.030 (0.78)	− 0.141 (0.18)	0.057 (0.59)	0.030 (0.78)	− 0.116 (0.28)
Joint attention	− 0.175 (0.10)	0.098 (0.36)	0.086 (0.42)	0.075 (0.48)	0.126 (0.24)	**− 0.283 (0.01)**
Moving toy	− 0.081 (0.45)	0.136 (0.20)	− 0.001 (0.99)	0.105 (0.33)	− 0.019 (0.86)	− 0.099 (0.35)

Cells contain Spearman partial correlation coefficients along with the corresponding two-sided *p* values in parentheses. The correlation coefficients are computed on the data of all individuals with ASD with participant’s age, sex and the KBIT-2 IQ composite score being used as covariates. Cells with *p* values below 0.05 are highlighted in bold

*n* indicates the number of participants, *ASD* autism spectrum disorder, *IQ* intelligence quotient, *KBIT-2* Kaufman Brief Intelligence Test-2

## Discussion

This study examined spontaneous visual attention to dynamic stimuli across specific experimental conditions in participants with and without ASD, aged 6–63 years. Attention to the screen, measured by % *Valid Time*, was above 85% on average in both the ASD and TD groups, suggesting good task attention overall. There was no significant difference between the two groups for attention to the screen between the four experimental conditions, and both groups showed similar patterns of visual attention allocation across ROIs in the four conditions. Specifically, in relation to social attention, there was no significant difference in average looking time at faces between the groups in the *Dyadic Bid* condition, contrary to what was previously reported in toddlers with ASD (Chawarska et al., 2012). Both ASD and TD groups allocated more visual attention to faces during this condition. However, unlike the findings in toddlers, significant differences in attention to faces were seen in the *Activity* and the *Joint Attention* experimental conditions. This indicates that while social attention is reduced in older children and adults with ASD, compared to TD groups, there are qualitative differences in the allocation of visual resources across the developmental trajectory of ASD. Differences in experimental conditions, or context, do not impact allocation of social attention in the same way across different age groups in ASD.

We also replicated findings that within general viewing of the face the ASD group paid less attention to eyes and mouths. However, similarly, this difference was only statistically significant in one specific experimental condition (*Activity* condition), where the ASD group paid less attention to the mouth area.

Consideration of the context in which patterns of eye gaze differ across age may help with understanding the mechanisms and developmental trajectory of social attention in ASD. The *Activity* condition was distributed throughout the duration of the video stimuli, but the vast majority of activity time was in the first portion of the videos. Individuals with ASD did attend to the faces during this time, but the TD group attended longer. It may be that the result of competing non-social factors, which were novel at the beginning of the videos, was that the ASD group did not prioritize attention to faces at the point at which they were first exploring the visual scene. This would be consistent with a previous report (Kwon et al., 2019) that hypothesized that the presence of distractors rather than attention to faces was a driving factor moderating differences in viewing. The authors suggest that competition between faces and external distractors might be a more robust measure than attention to faces itself.

Salience of non-social stimuli may also explain the differences seen in the *Joint Attention* condition. This experimental condition always preceded the *Moving Toy* condition, in which the actor was looking at a static toy while the same toy as previously was moving. Therefore, the novelty of the toy moving in the *Joint Attention* condition might have been more salient and attracted more attention from individuals with ASD. In the second condition with the moving toy, the actors’ face may have not had the same competition for salience and therefore no differences were observed.

Unlike the studies in toddlers (Campbell et al., 2014; Chawarska et al., 2012), the *Dyadic Bid* condition did not reveal any differences in attention to faces. This may be a result of developmental changes in one or both groups, either with experience or training or both. For example, improvements in processing of social information in TD individuals may mean that less attention is given to faces, as pertinent information can be garnered in a shorter time (i.e., they infer more information from faces more quickly). In contrast, some individuals with ASD may have learned or been specifically taught the relevance of attention to looking at a person who is talking to them, thus resulting in longer gaze time. Alternatively, increased efficiency of social attention in the TD group may not be picked up when using ROIs to analyze the eye-tracking signal. This hypothesis requires additional eye-tracking features to determine whether focus on the amount of viewing time might have masked other differences potentially existing between the two groups. Some evidence comes from a time-course analysis study that showed no difference in viewing time for faces between the ASD and TD groups but found that the TD group was quicker to look first and then look less as time went on (Freeth et al., 2010). Other studies with older individuals with ASD also indicate that although total looking time may be the same, differences may be found in the timing and slower response to socially informative elements in the TD group (Caruana et al., 2018; Fletcher-Watson et al., 2009; Frost-Karlsson et al., 2019). In terms of impact on social functioning, less urgency to look to faces might mean that important information is missed.

The *Activity* condition revealed more looking at the mouth by the TD group, which was also the reverse of the results observed in toddlers, where mouth viewing has been related to poor outcomes in the ASD group (Chawarska et al., 2012). Here, however, in older children and adults, mouth viewing may be an effective strategy that has developed in TD individuals—scanning of face and attention to mouth in anticipation of verbal communication. In contrast, studies comparing ASD and TD adults in both real-life or online Skype situations have found increased attention to mouths by ASD participants in certain contexts, for example when discussion relates to emotional factors (Hutchins & Brien, 2016). The relationship between attention, as manifested in *% looking time*, to the core features of faces is complex in ASD. A multitude of bottom-up and top-down processes may potentially influence the allocation of social attention in both groups, and it is likely that these become more sensitive to modulation by context with development and learning between toddlerhood and older child and adulthood.

Previous studies reported significant relationships between severity of ASD symptoms, as captured by a variety of behavior rating scales, and eye-tracking based measurements (Frazier et al., 2018; Murias et al., 2018). We hypothesized that similar relationships could have been established in our data. However, only a few relationships, not many more than would be expected by chance, were identified. A recent comparative study also did not report substantial relationships between behavior rating scales and eye-tracking behavior (Fujioka et al., 2020). This lack of consistent finding of relationships may be due to, as the authors propose, the scale measures capturing a wide range of social deficits, of which eye looking or contact is only one. Further, it could be that allocation of social attention, measured by eye-tracking, is a different construct to that captured in behavioral report and provides something additional to assessment of social attention. In the context of biosensors such as eye-tracking for use in clinical trials it is important to compare how both direct and behavioral report measures change over time to determine whether one may be more sensitive as an outcome measure than the other, and also whether short-term biosensor changes may lead to longer term changes in observable behavior as reported in scales.

The purpose of this study was to investigate the use of a paradigm that has been well established in one age group (toddlers) in the literature (Chawarska et al., 2012, 2013, 2016; Wang et al., 2018) in a group of older individuals with ASD in order to explore its potential as a biomarker in clinical trials. Often the paradigms designed to measure social attention typically minimize the number of examined parameters in order to facilitate comparison between the ASD and TD groups. As a consequence, a possible limitation is that this increasingly results in a situation when a tested paradigm does not resemble a real-life social interaction. In particular, the paradigm tested in the current study was developed to be suitable for younger children. Although we were able to establish persistent differences in social attention between an older group of TD controls and ASD individuals, the tested paradigm in older children and adults may be less reflective of a real-life social interaction than it may be for toddlers (Macari et al., 2021). Therefore, the differences observed may be anomalies of the paradigm rather than typical viewing patterns of either group in real-life (Grossman et al., 2019; Hanley et al., 2015; Risko et al., 2016). Furthermore, by combining the response to both naturalistic and experimentally controlled or probe paradigms might lead to more helpful characterizations of social attention in ASD that could help with subtyping for clinical trials, similar to the approach adopted for the development of the Autism Risk Index (Frazier et al., 2018). In addition, albeit helpful and important in replication of other eye-tracking studies, the use of ROIs may mask other differences in features that may be more consistent across ASD development, could be more closely related to ASD symptoms, or more sensitive to change. Such additional features could reflect scan-path length and recursion during exploration of a visual scene as well as fixation rate (Heaton & Freeth, 2016), duration and frequency of saccades (Vabalas & Freeth, 2016), and variability of gaze patterns (Avni et al., 2020). Future studies should include these features to ensure that differences and similarities in viewing patterns are captured and characterized more fully.

In conclusion, the present findings support the general observation that eye-tracking studies using ROI can demonstrate differences in allocation of visual resources to social scenes in individuals with ASD compared to TD groups. However, the differences in allocation are context specific, and, depending on the experimental condition, there may be no differences between the groups, or the results could differ by age of the participants. Studies such as this can contribute to our understanding of the developmental trajectory of social attention in ASD, as well as support the process of identification of biomarkers for clinical trials. For utility of eye-tracking and social attention as a potential biomarker we need to select paradigms that include context and probes that have been shown to detect differences in the target age group. In addition, we should consider features of eye-tracking beyond ROI that may capture more information about the qualitative differences in response to social stimuli.

## Supplementary Information

Below is the link to the electronic supplementary material.

Supplementary file1 (PDF 481 KB)

## Data Availability

The data sharing policy of Janssen Pharmaceutical Companies of Johnson & Johnson is available at https://www.janssen.com/clinical-trials/transparency. Requests for access to the study data can be submitted through Yale Open Data Access (YODA) Project site at http://yoda.yale.edu.

## References

[CR1] Aman MG, Novotny S, Samango-Sprouse C, Lecavalier L, Leonard E, Gadow KD, King BH, Pearson DA, AnnGernsbacher M, Chez M (2004). Outcome measures for clinical drug trials in autism. CNS Spectrums.

[CR2] Aman MG, Singh NN (2017). Aberrant behavior checklist manual.

[CR3] Avni I, Meiri G, Bar-Sinai A, Reboh D, Manelis L, Flusser H, Michaelovski A, Menashe I, Dinstein I (2020). Children with autism observe social interactions in an idiosyncratic manner. Autism Research.

[CR4] Bangerter A, Chatterjee M, Manfredonia J, Manyakov NV, Ness S, Boice MA, Skalkin A, Goodwin MS, Dawson G, Hendren R, Leventhal B, Shic F, Pandina G (2020). Automated recognition of spontaneous facial expression in individuals with autism spectrum disorder: Parsing response variability. Molecular Autism.

[CR5] Bangerter A, Chatterjee M, Manyakov NV, Ness S, Lewin D, Skalkin A, Boice M, Goodwin MS, Dawson G, Hendren R, Leventhal B, Shic F, Esbensen A, Pandina G (2020). Relationship between sleep and behavior in autism spectrum disorder: Exploring the impact of sleep variability. Frontiers in Neuroscience.

[CR6] Bangerter A, Ness S, Aman MG, Esbensen AJ, Goodwin MS, Dawson G, Hendren R, Leventhal B, Khan A, Opler M, Harris A, Pandina G (2017). Autism behavior inventory: A novel tool for assessing core and associated symptoms of autism spectrum disorder. Journal of Child and Adolescent Psychopharmacology.

[CR7] Baron-Cohen S, Wheelwright S, Hill J, Raste Y, Plumb I (2001). The “reading the mind in the eyes” test revised version: A study with normal adults, and adults with Asperger syndrome or high-functioning autism. Journal of Child Psychology and Psychiatry.

[CR8] Blignaut P, Wium D (2014). Eye-tracking data quality as affected by ethnicity and experimental design. Behavior Research Methods.

[CR9] Campbell DJ, Shic F, Macari S, Chawarska K (2014). Gaze response to dyadic bids at 2 years related to outcomes at 3 years in autism spectrum disorders: A subtyping analysis. Journal of Autism and Developmental Disorders.

[CR10] Caruana N, Stieglitz Ham H, Brock J, Woolgar A, Kloth N, Palermo R, McAethur G (2018). Joint attention difficulties in autistic adults: An interactive eye-tracking study. Autism.

[CR11] Chawarska K, Macari S, Shic F (2012). Context modulates attention to social scenes in toddlers with autism. Journal of Child Psychology and Psychiatry.

[CR12] Chawarska K, Macari S, Shic F (2013). Decreased spontaneous attention to social scenes in 6-month-old infants later diagnosed with autism spectrum disorders. Biological Psychiatry.

[CR13] Chawarska K, Ye S, Shic F, Chen L (2016). Multilevel differences in spontaneous social attention in toddlers with autism spectrum disorder. Child Development.

[CR14] Chevallier C, Parish-Morris J, McVey A, Rump KM, Sasson NJ, Herrington JD, Schultz RT (2015). Measuring social attention and motivation in autism spectrum disorder using eye-tracking: Stimulus type matters. Autism Research.

[CR15] Chita-Tegmark M (2016). Social attention in ASD: A review and meta-analysis of eye-tracking studies. Research in Developmental Disabilities.

[CR16] Constantino JN, Davis SA, Todd RD, Schindler MK, Gross MM, Brophy SL, Metzger LM, Shoushtari CS, Splinter R, Reich W (2003). Validation of a brief quantitative measure of autistic traits: Comparison of the social responsiveness scale with the autism diagnostic interview-revised. Journal of Autism and Developmental Disorders.

[CR17] Dawson G, Meltzoff AN, Osterling J, Rinaldi J, Brown E (1998). Children with autism fail to orient to naturally occurring social stimuli. Journal of Autism and Developmental Disorders.

[CR18] Del Bianco T, Mazzoni N, Bentenuto A, Venuti P (2018). An investigation of attention to faces and eyes: Looking time is task-dependent in autism spectrum disorder. Frontiers in Psychology.

[CR19] Fletcher-Watson S, Leekam SR, Benson V, Frank MC, Findlay JM (2009). Eye-movements reveal attention to social information in autism spectrum disorder. Neuropsychologia.

[CR20] Fox J, Weisberg S (2011). An R companion to applied regression.

[CR21] Frazier TW, Klingemier EW, Parikh S, Speer L, Strauss MS, Eng C, Harden AY, Youngstrom EA (2018). Development and validation of objective and quantitative eye tracking-based measures of autism risk and symptom levels. Journal of the American Academy of Child and Adolescent Psychiatry.

[CR22] Frazier TW, Strauss M, Klingemier EW, Zetzer EE, Hardan AY, Eng C, Youngstrom EA (2017). A meta-analysis of gaze differences to social and nonsocial information between individuals with and without autism. Journal of the American Academy of Child and Adolescent Psychiatry.

[CR23] Freeth M, Bugembe P (2019). Social partner gaze direction and conversational phase; factors affecting social attention during face-to-face conversations in autistic adults?. Autism.

[CR24] Freeth M, Chapman P, Ropar D, Mitchell P (2010). Do gaze cues in complex scenes capture and direct the attention of high functioning adolescents with ASD? Evidence from eye-tracking. Journal of Autism and Developmental Disorders.

[CR25] Frischen A, Bayliss AP, Tipper SP (2007). Gaze cueing of attention: Visual attention, social cognition, and individual differences. Psychological Bulletin.

[CR26] Frost-Karlsson M, Galazka MA, Gillberg C, Gillberg C, Miniscalco C, Billstedt E, Hadjikhani N, Åsberg Johnels J (2019). Social scene perception in autism spectrum disorder: An eye-tracking and pupillometric study. Journal of Clinical and Experimental Neuropsychology.

[CR27] Fujioka T, Tsuchiya KJ, Saito M, Hirano Y, Matsuo M, Kikuchi M, Maegaki Y, Choi D, Kato S, Yoshida T, Yoshimura Y, Ooba S, Mizuno Y, Takiguchi S, Matsuzaki H, Tomoda A, Shudo K, Ninomiya M, Katayama T, Kosaka H (2020). Developmental changes in attention to social information from childhood to adolescence in autism spectrum disorders: A comparative study. Molecular Autism.

[CR28] Gadow K, Sprafkin J (1997). Early childhood inventory-4 norms manual.

[CR29] Grossman RB, Zane E, Mertens J, Mitchell T (2019). Facetime vs. Screentime: Gaze patterns to live and video social stimuli in adolescents with ASD. Scientific Reports.

[CR30] Guillon Q, Hadjikhani N, Baduel S, Roge B (2014). Visual social attention in autism spectrum disorder: Insights from eye tracking studies. Neuroscience and Biobehavioral Reviews.

[CR31] Hanley M, Riby DM, Carty C, Melaugh McAteer A, Kennedy A, McPhillips M (2015). The use of eye-tracking to explore social difficulties in cognitively able students with autism spectrum disorder: A pilot investigation. Autism.

[CR32] Heaton TJ, Freeth M (2016). Reduced visual exploration when viewing photographic scenes in individuals with autism spectrum disorder. Journal of Abnormal Psychology.

[CR33] Hessels R, Holleman G, Cornelissen T, Hooge I, Kemner C (2018). Eye contact takes two—Autistic and social anxiety traits predict gaze behavior in dyadic interaction. Journal of Experimental Psychopathology.

[CR34] Hirose Y, Kennedy A, Tatler BW (2010). Perception and memory across viewpoint changes in moving images. Journal of Vision.

[CR35] Hutchins T, Brien A (2016). Conversational topic moderates social attention in autism spectrum disorder: Talking about emotions is like driving in a snowstorm. Research in Autism Spectrum Disorders.

[CR36] Jagannatha S, Sargsyan D, Manyakov N, Skalkin A, Bangerter A, Ness S, Lewin D, Johnson K, Durham K, Pandina G (2019). A practical application of data mining methods to build predictive models for autism spectrum disorder based on biosensor data from Janssen autism knowledge engine (JAKE®). Statistics in Biopharmaceutical Research.

[CR37] Jones W, Klin A (2013). Attention to eyes is present but in decline in 2–6-month-old infants later diagnosed with autism. Nature.

[CR38] Kaufman A, Kaufman N (2004). Kaufman brief intelligence test.

[CR39] Kim S (2015). ppcor: An R package for a fast calculation to semi-partial correlation coefficients. Communications for Statistical Applications and Methods.

[CR40] Klin A, Jones W, Schultz R, Volkmar F, Cohen D (2002). Visual fixation patterns during viewing of naturalistic social situations as predictors of social competence in individuals with autism. Archives of General Psychiatry.

[CR41] Kwon MK, Moore A, Barnes CC, Cha D, Pierce K (2019). Typical levels of eye-region fixation in toddlers with autism spectrum disorder across multiple contexts. Journal of the American Academy of Child and Adolescent Psychiatry.

[CR42] Lam KS, Aman MG (2007). The repetitive behavior scale-revised: Independent validation in individuals with autism spectrum disorders. Journal of Autism and Developmental Disorders.

[CR43] Lenth R (2016). Least-squares means: The R package lsmeans. Journal of Statistical Software.

[CR44] Lord C, Luyster R, Gotham K, Guthrie W (2012). Autism Diagnostic Observation Schedule, Second Edition (ADOS-2).

[CR45] Macari S, Milgramm A, Reed J, Shic F, Powell KK, Macris D, Chawarska K (2021). Context-specific dyadic attention vulnerabilities during the first year in infants later developing autism spectrum disorder. Journal of the American Academy of Child and Adolescent Psychiatry.

[CR46] Manfredonia J, Bangerter A, Manyakov NV, Ness S, Lewin D, Skalkin A, Boice M, Goodwin MS, Dawson G, Hendren R, Leventhal B, Shic F, Pandina G (2019). Automatic recognition of posed facial expression of emotion in individuals with autism spectrum disorder. Journal of Autism and Developmental Disorders.

[CR47] Manyakov NV, Bangerter A, Chatterjee M, Mason L, Ness S, Lewin D, Skalkin A, Boice M, Goodwin MS, Dawson G, Hendren R, Leventhal B, Shic F, Pandina G (2018). Visual exploration in autism spectrum disorder: Exploring age differences and dynamic features using recurrence quantification analysis. Autism Research.

[CR48] Murias M, Major S, Davlantis K, Franz L, Harris A, Rardin B, Sabatos-DeVito M, Dawson G (2018). Validation of eye-tracking measures of social attention as a potential biomarker for autism clinical trials. Autism Research.

[CR49] Ness SL, Manyakov NV, Bangerter A, Lewin D, Jagannatha S, Boice M, Skalkin A, Dawson G, Janvier YM, Goodwin MS, Hendren R, Leventhal B, Shic F, Cioccia W, Pandina G (2017). JAKE(R) multimodal data capture system: Insights from an observational study of autism spectrum disorder. Frontiers in Neuroscience.

[CR50] Peterson MF, Eckstein MP (2012). Looking just below the eyes is optimal across face recognition tasks. Proceeding of the National Academy of Sciences USA.

[CR51] Pinheiro, J., Bates, D., DebRoy, S., & Sarkar, D. (2018). nlme: linear and nonlinear mixed effects models. R package version 3.1–137. Retrieved from https://CRAN.R-project.org/package=nlme

[CR52] Plesa Skwerer D, Brukilacchio B, Chu A, Eggleston B, Meyer S, Tager-Flusberg H (2019). Do minimally verbal and verbally fluent individuals with autism spectrum disorder differ in their viewing patterns of dynamic social scenes?. Autism.

[CR53] Risko E, Richardson D, Kingstone A (2016). Breaking the fourth wall of cognitive science: Real-world social attention and the dual function of gaze. Current Directions in Psychological Science.

[CR54] Rutter M, Bailey A, Lord C (2003). Social communication questionnaire.

[CR55] Sargsyan D, Jagannatha S, Manyakov N, Skalkin A, Bangerter A, Ness S (2017). Feature selection with weighted importance index in an autism spectrum disorder study. Statistics in Biopharmaceutical Research.

[CR56] Senju A, Csibra G, Johnson MH (2008). Understanding the referential nature of looking: Infants’ preference for object-directed gaze. Cognition.

[CR57] Sheehan DV, Lecrubier Y, Sheehan KH, Amorim P, Janavs J, Weiller E, Hergueta T, Baker R, Dunbar GC (1998). The mini-international neuropsychiatric interview (MINI): The development and validation of a structured diagnostic psychiatric interview for DSM-IV and ICD-10. Journal of Clinical Psychiatry.

[CR58] Sheehan DV, Sheehan KH, Shytle RD, Janavs J, Bannon Y, Rogers JE, Milo KM, Stock SL, Wilkinson B (2010). Reliability and validity of the mini international neuropsychiatric interview for children and adolescents (MINI-KID). Journal of Clinical Psychiatry.

[CR59] Vabalas A, Freeth M (2016). Brief report: Patterns of eye movements in face to face conversation are associated with autistic traits: Evidence from a student sample. Journal of Autism and Developmental Disorders.

[CR60] Wang Q, Campbell DJ, Macari SL, Chawarska K, Shic F (2018). Operationalizing atypical gaze in toddlers with autism spectrum disorders: A cohesion-based approach. Molecular Autism.

