# Supplementary material for: Context Modulates Attention to Faces in Dynamic Social Scenes in Children and Adults with Autism Spectrum Disorder
Source: J Autism Dev Disord. 2021 Oct 8;52(10):4219–32. doi: 10.1007/s10803-021-05279-z (PMC9508054; doi:10.1007/s10803-021-05279-z)
Supplement: Supplementary file 1 — Supplementary file1 (PDF 481 KB) [file 10803_2021_5279_MOESM1_ESM.pdf]

Supplementary Information

Supplementary Table 1. The order of episodes of different stimulus conditions in each video stimulus.

Episode no.	Video with a *female* actor		Video with a *male* actor	
	Time interval, sec	Stimulus condition	Time interval, sec	Stimulus condition
1	00 – 22	Activity	00–29	Activity
2	22 – 27	Dyadic Bid	29–36	Dyadic Bid
3	27 – 31	Activity	36–40	Activity
4	31 – 40	Joint Attention	40–46	Joint Attention
5	40 – 46	Activity	46–50	Dyadic Bid
6	46 – 49	Dyadic Bid	50–57	Activity
7	45 – 55	Activity	57–62	Moving Toy
8	55 – 60	Moving Toy	62–67	Dyadic Bid
9	60 – 67	Activity	67–90	Activity
10	67 – 73	Dyadic Bid	-	-

Time intervals are aligned with respect to stimulus onset

Supplementary Table 2. Fixed effects in the model of % *Valid Time*.

Fixed effect	χ^2-statistic	df	*P*-value
Intercept	1076.04	1	**$< 10^{-15}$**
Participant group	1.41	1	0.24
Participant's age	2.51	1	0.11
Participant's sex	0.96	1	0.33
Stimulus condition	20.24	3	**$< 5 \cdot 10^{-4}$**
Stimulus condition x Participant group	13.68	3	**$< 5 \cdot 10^{-3}$**

Significance of the fixed effects is tested using the analysis of variance type III sum of squares and the Wald χ^2 test. P-values below 0.05 are highlighted in bold. df, degrees of freedom

Supplementary Table 3. Post-hoc pair-wise comparisons in the model of % *Valid Time*.

Comparison	Estimate	SE	df	*t*-statistic	*P*-value
ASD, Activity vs. TD, Activity	−2.56	2.16	128	−1.19	0.93
ASD, Activity vs. ASD, Dyadic Bid	−1.09	0.43	2502	−2.54	0.18
ASD, Activity vs. TD, Dyadic Bid	−5.06	2.16	128	−2.35	0.28
ASD, Activity vs. ASD, Joint Attention	−1.67	0.43	2502	−3.89	**$< 5 \cdot 10^{-3}$**
ASD, Activity vs. TD, Joint Attention	−2.73	2.16	128	−1.27	0.91
ASD, Activity vs. ASD, Moving Toy	−1.67	0.43	2502	−3.90	**$< 5 \cdot 10^{-3}$**
ASD, Activity vs. TD, Moving Toy	−4.63	2.16	128	−2.15	0.39
TD, Activity vs. ASD, Dyadic Bid	1.47	2.16	128	0.68	1.00
TD, Activity vs. TD, Dyadic Bid	−2.50	0.68	2502	−3.71	**0.01**
TD, Activity vs. ASD, Joint Attention	0.89	2.16	128	0.41	1.00
TD, Activity vs. TD, Joint Attention	−0.17	0.68	2502	−0.26	1.00
TD, Activity vs. ASD, Moving Toy	0.89	2.16	128	0.41	1.00
TD, Activity vs. TD, Moving Toy	−2.07	0.68	2502	−3.07	**0.04**
ASD, Dyadic Bid vs. TD, Dyadic Bid	−3.97	2.16	128	−1.84	0.59
ASD, Dyadic Bid vs. ASD, Joint Attention	−0.58	0.43	2502	−1.35	0.88
ASD, Dyadic Bid vs. TD, Joint Attention	−1.64	2.16	128	−0.76	0.99
ASD, Dyadic Bid vs. ASD, Moving Toy	−0.58	0.43	2502	−1.36	0.88
ASD, Dyadic Bid vs. TD, Moving Toy	−3.54	2.16	128	−1.64	0.72
TD, Dyadic Bid vs. ASD, Joint Attention	3.39	2.16	128	1.57	0.76
TD, Dyadic Bid vs. TD, Joint Attention	2.33	0.68	2502	3.45	**0.01**
TD, Dyadic Bid vs. ASD, Moving Toy	3.39	2.16	128	1.57	0.77
TD, Dyadic Bid vs. TD, Moving Toy	0.43	0.68	2502	0.64	1.00
ASD, Joint Attention vs. TD, Joint Attention	−1.06	2.16	128	−0.49	1.00
ASD, Joint Attention vs. ASD, Moving Toy	−0.002	0.43	2502	−0.01	1.00
ASD, Joint Attention vs. TD, Moving Toy	−2.96	2.16	128	−1.38	0.87
TD, Joint Attention vs. ASD, Moving Toy	1.06	2.16	128	0.49	1.00
TD, Joint Attention vs. TD, Moving Toy	−1.90	0.68	2502	−2.82	0.09
ASD, Moving Toy vs. TD, Moving Toy	−2.96	2.16	128	−1.37	0.87

ASD, autism spectrum disorder; df, degrees of freedom; SE, standard error; TD, typically developing

Supplementary Table 4. Least-squares mean estimates and 95% two-sided confidence intervals for different levels of the categorical factors in the model of % *Valid Time*.

	Condition		Activity	Dyadic Bid	Joint Attention	Moving Toy
Group	n	Statistic				
ASD	94	Mean	85.3	86.4	87.0	87.0
	94	CI	82.9–87.8	84.0–88.9	84.5–89.4	84.5–89.5
TD	38	Mean	87.9	90.4	88.1	90.0
	38	CI	84.2–91.5	86.7–94.1	84.4–91.7	86.3–93.6

'n' indicates the number of participants. ASD, autism spectrum disorder; CI, confidence interval; TD, typically developing

Supplementary Table 5. Fixed effects in the models of *% looking time* for different stimulus conditions incorporating the five regions of interest.

Stimulus condition	Fixed effect	χ^2-statistic	df	*P*-value
Activity	Intercept	16.50	1	**$< 10^{-4}$**
	Participant group	0.33	1	0.56
	Participant's age	0.00	1	1.00
	Participant's sex	0.00	1	1.00
	Region-of-interest	3342.93	4	**$< 10^{-15}$**
	Region-of-interest x Participant group	15.33	4	**$< 5 \cdot 10^{-3}$**
Dyadic Bid	Intercept	7.50	1	**0.01**
	Participant group	0.42	1	0.52
	Participant's age	0.00	1	1.00
	Participant's sex	0.00	1	1.00
	Region-of-interest	1532.74	4	**$< 10^{-15}$**
	Region-of-interest x Participant group	11.24	4	**0.02**
Joint Attention	Intercept	12.95	1	**$< 5 \cdot 10^{-4}$**
	Participant group	0.11	1	0.74
	Participant's age	0.00	1	1.00
	Participant's sex	0.00	1	1.00
	Region-of-interest	766.15	1	**$< 10^{-15}$**
	Region-of-interest x Participant group	28.45	4	**$< 10^{-4}$**
Moving Toy	Intercept	3.67	1	0.06
	Participant group	0.33	1	0.57
	Participant's age	0.00	1	1.00
	Participant's sex	0.00	1	1.00
	Region-of-interest	1165.11	4	**$< 10^{-15}$**
	Region-of-interest x Participant group	1.05	4	0.90

The data of each stimulus condition are modelled separately. The same five regions of interest are analyzed as those presented in ***Figure 2****. Significance of the fixed effects is assessed using the analysis of variance type III sum of squares and the Wald χ^2 test. P-values below 0.05 are in bold. df, degrees of freedom*

Supplementary Table 6. Least-squares mean estimates and 95% two-sided confidence intervals for different levels of the categorical factors in the models of *% looking time* for different stimulus conditions incorporating the five regions of interest.

Condition	Group	ROI / Statistic	Background	Body	Hands/ Activity	Face	Toys
Activity	ASD (n = 94)	Mean	3.71	3.89	47.93	30.72	13.74
		CI	2.38–5.04	2.56–5.22	46.61–49.26	29.39–32.05	12.41–15.07
	TD (n = 38)	Mean	2.99	3.56	46.31	34.91	12.20
		CI	0.93–5.06	1.50–5.63	44.24–48.37	32.85–36.98	10.13–14.27
Dyadic Bid	ASD (n = 94)	Mean	3.95	4.61	33.78	49.26	8.38
		CI	1.85–6.06	2.51–6.72	31.67–35.88	47.16–51.36	6.28–10.49
	TD (n = 38)	Mean	2.69	3.90	39.39	48.58	5.44
		CI	−0.59–5.96	0.63–7.17	36.12–42.66	45.31–51.85	2.17–8.71
Joint Attention	ASD (n = 94)	Mean	5.38	3.16	36.51	24.53	30.40
		CI	3.20–7.56	0.99–5.34	34.33–38.69	22.35–26.71	28.22–32.58
	TD (n = 38)	Mean	4.71	3.07	34.63	33.20	24.35
		CI	1.32–8.09	−0.32–6.46	31.25–38.02	29.81–36.59	20.96–27.74
Moving Toy	ASD (n = 94)	Mean	3.39	2.74	12.91	25.51	55.44
		CI	0.82–5.97	0.16–5.31	10.33–15.49	22.93–28.09	52.86–58.01
	TD (n = 38)	Mean	2.02	3.10	14.00	26.65	54.23
		CI	−1.99–6.03	−0.91–7.11	10.00–18.01	22.64–30.65	50.22–58.23

The data are obtained using the same linear−mixed effects models as those listed in ***Supplementary Table 5****. 'n' indicates the number of participants. ASD, autism spectrum disorder; CI, confidence interval; ROI, region-of-interest; TD, typically developing*

Supplementary Table 7. Fixed effects in the models of *% looking time* for different stimulus conditions incorporating the core features of faces as regions of interest.

Stimulus condition	Fixed effect	χ^2-statistic	df	*P*-value
Activity	Intercept	42.61	1	**$< 10^{-10}$**
	Participant group	0.78	1	0.38
	Participant's age	1.18	1	0.28
	Participant's sex	0.00	1	0.99
	Region-of-interest	1209.00	2	**$< 10^{-15}$**
	Region-of-interest x Participant group	9.70	2	**0.01**
Dyadic Bid	Intercept	136.01	1	**$< 10^{-15}$**
	Participant group	0.05	1	0.83
	Participant's age	0.05	1	0.83
	Participant's sex	2.28	1	0.13
	Region-of-interest	151.54	2	**$< 10^{-15}$**
	Region-of-interest x Participant group	1.39	2	0.50
Joint Attention	Intercept	54.76	1	**$< 10^{-12}$**
	Participant group	3.62	1	0.06
	Participant's age	0.63	1	0.43
	Participant's sex	0.03	1	0.86
	Region-of-interest	22.18	2	**$< 10^{-4}$**
	Region-of-interest x Participant group	1.64	2	0.44
Moving Toy	Intercept	43.63	1	**$< 10^{-10}$**
	Participant group	0.94	1	0.33
	Participant's age	0.23	1	0.63
	Participant's sex	0.03	1	0.86
	Region-of-interest	38.68	2	**$< 10^{-8}$**
	Region-of-interest x Participant group	2.68	2	0.26

The data of each stimulus conditions are modelled separately. The same three regions of interest are analyzed as those presented in ***Figure 3****. Significance of the fixed effects is assessed using the analysis of variance type III sum of squares and the Wald* χ^2 *test. P-values below 0.05 are in bold. df, degrees of freedom*

Supplementary Table 8. Least-squares mean estimates and 95% two-sided confidence intervals for different levels of the categorical factors in the models of *% looking time* for different stimulus conditions incorporating the core features of faces as regions of interest.

Condition	Group	ROI / Statistic	Eyes	Mouth	Hair
Activity	ASD (n = 94)	Mean	4.89	24.70	1.08
		CI	3.84–5.93	23.65–25.75	0.03–2.13
	TD (n = 38)	Mean	5.74	28.53	0.80
		CI	4.12–7.36	26.91–30.14	−0.82–2.42
Dyadic Bid	ASD (n = 94)	Mean	24.84	19.51	3.52
		CI	22.24–27.43	16.92–22.10	0.93–6.11
	TD (n = 38)	Mean	25.35	20.97	1.18
		CI	21.35–29.36	16.97–24.98	−2.82–5.18
Joint Attention	ASD (n = 94)	Mean	11.53	7.89	5.30
		CI	9.62–13.45	5.98–9.80	3.39–7.22
	TD (n = 38)	Mean	14.88	11.91	6.31
		CI	11.92–17.83	8.96–14.87	3.35–9.26
Moving Toy	ASD (n = 94)	Mean	10.53	11.48	3.67
		CI	8.55–12.52	9.50–13.47	1.68–5.65
	TD (n = 38)	Mean	12.29	12.74	1.58
		CI	9.24–15.35	9.68–15.80	−1.48–4.64

The data are obtained using the same linear-mixed effects models as those listed in ***Supplementary Table 7****. 'n' indicates the number of participants. ASD, autism spectrum disorder; CI, confidence interval; ROI, region-of-interest; TD, typically developing.*

Supplementary Table 9. Spearman correlations between % *Valid Time*, *% looking time* for different regions of interest and the KBIT-2 IQ composite score in groups of the individuals with ASD with varying levels of IQ.

Stimulus condition			Activity	Dyadic Bid	Joint Attention	Moving Toy
Region-of-interest	IQ range	n				
% Valid Time	all	94	−0.029 (0.78)	0.089 (0.39)	0.106 (0.31)	−0.056 (0.59)
	60–84	21	0.219 (0.34)	0.101 (0.66)	0.386 (0.08)	0.417 (0.06)
	85–115	54	0.011 (0.93)	**0.280 (0.04)**	0.094 (0.50)	0.028 (0.84)
	116–136	19	0.063 (0.80)	0.219 (0.37)	−0.032 (0.90)	0.116 (0.64)
Background	all	94	0.159 (0.13)	0.145 (0.16)	−0.028 (0.79)	0.043 (0.68)
	60–84	21	0.138 (0.55)	0.032 (0.89)	0.056 (0.81)	0.226 (0.32)
	85–115	54	−0.215 (0.12)	−0.041 (0.77)	−0.166 (0.23)	−0.162 (0.24)
	116–136	19	0.301 (0.21)	0.074 (0.76)	0.277 (0.25)	0.197 (0.42)
Body	all	94	−0.169 (0.10)	−0.081 (0.44)	−0.063 (0.55)	0.010 (0.92)
	60–84	21	−0.197 (0.39)	0.092 (0.69)	−0.126 (0.59)	0.130 (0.58)
	85–115	54	0.002 (0.99)	−0.187 (0.18)	−0.001 (0.99)	0.102 (0.46)
	116–136	19	0.358 (0.13)	−0.003 (0.99)	0.054 (0.83)	0.410 (0.08)
Hands/Activity	all	94	0.028 (0.79)	−0.099 (0.34)	0.025 (0.81)	−0.153 (0.14)
	60–84	21	−0.107 (0.65)	−0.172 (0.46)	−0.141 (0.54)	−0.178 (0.44)
	85–115	54	0.213 (0.12)	0.163 (0.24)	0.157 (0.26)	0.165 (0.23)
	116–136	19	−0.254 (0.29)	−0.124 (0.61)	0.082 (0.74)	0.139 (0.57)
Face	all	94	0.032 (0.76)	0.032 (0.76)	0.103 (0.32)	−0.018 (0.86)
	60–84	21	−0.021 (0.93)	0.111 (0.63)	−0.046 (0.84)	−0.351 (0.12)
	85–115	54	−0.026 (0.85)	−0.179 (0.20)	0.070 (0.62)	−0.102 (0.47)
	116–136	19	0.282 (0.24)	0.058 (0.81)	−0.123 (0.62)	0.072 (0.77)
Eyes	all	94	−0.061 (0.56)	−0.055 (0.60)	0.016 (0.88)	−0.053 (0.61)
	60–84	21	−0.114 (0.62)	−0.077 (0.74)	0.320 (0.16)	−0.335 (0.14)
	85–115	54	−0.244 (0.08)	−0.232 (0.09)	−0.100 (0.47)	−0.084 (0.54)
	116–136	19	−0.053 (0.83)	−0.143 (0.56)	−0.123 (0.62)	−0.112 (0.65)
Mouth	all	94	0.015 (0.89)	0.049 (0.64)	0.033 (0.75)	−0.053 (0.61)
	60–84	21	0.275 (0.23)	0.347 (0.12)	−0.057 (0.81)	−0.049 (0.83)
	85–115	54	0.092 (0.51)	0.222 (0.11)	0.245 (0.07)	−0.004 (0.98)
	116–136	19	0.270 (0.26)	0.294 (0.22)	−0.216 (0.37)	0.070 (0.77)
Hair	all	94	0.068 (0.52)	0.062 (0.55)	0.148 (0.16)	0.050 (0.64)

	60–84	21	−0.061 (0.79)	0.147 (0.52)	−0.213 (0.36)	0.148 (0.52)
	85–115	54	−0.127 (0.36)	−0.135 (0.33)	−0.028 (0.84)	−0.243 (0.08)
	116–136	19	0.154 (0.53)	0.212 (0.38)	0.229 (0.35)	0.147 (0.55)
Toys	all	94	0.017 (0.87)	0.041 (0.69)	−0.058 (0.58)	0.074 (0.48)
	60–84	21	0.197 (0.39)	−0.147 (0.52)	−0.165 (0.47)	**0.482 (0.03)**
	85–115	54	−0.135 (0.33)	0.011 (0.93)	−0.117 (0.40)	−0.040 (0.78)
	116–136	19	−0.363 (0.13)	−0.042 (0.86)	−0.061 (0.81)	−0.261 (0.28)

The data are presented for each stimulus condition and region-of-interest separately. Cells contain Spearman correlation coefficients, with the corresponding two-sided P-values being provided in parentheses. Cells with P-values below 0.05 are highlighted in bold (see also ***Supplementary Figure 2****). 'all' in column 'IQ range' refers to the whole ASD sample regardless of IQ level. 'n' indicates the number of participants. ASD, autism spectrum disorder; IQ, intelligence quotient; KBIT-2, Kaufman Brief Intelligence Test-2.*

Supplementary Table 10. Correlations between % *Valid Time*, *% looking time* for different regions of interest and severity of ASD symptoms.

Region-of-interest		% Valid Time	Background	Body	Hands/Activity	Face	Toys
ASD symptom	Condition						
Autism Behavior Inventory (n = 93)							
Core ASD symptom scale score (total score)	Activity	−0.205 (0.05)	0.147 (0.17)	0.179 (0.09)	−0.081 (0.45)	0.067 (0.53)	−0.081 (0.45)
	Dyadic Bid	−0.146 (0.17)	0.117 (0.27)	0.014 (0.89)	0.013 (0.90)	−0.056 (0.60)	−0.016 (0.88)
	Joint Attention	**−0.250 (0.02)**	0.158 (0.14)	0.080 (0.45)	−0.023 (0.83)	0.190 (0.07)	**−0.261 (0.01)**
	Moving Toy	**−0.225 (0.03)**	**0.265 (0.01)**	−0.020 (0.85)	0.027 (0.80)	0.054 (0.61)	−0.148 (0.16)
Challenging behavior	Activity	−0.178 (0.09)	−0.008 (0.94)	0.129 (0.23)	−0.008 (0.94)	0.067 (0.53)	−0.131 (0.22)
	Dyadic Bid	−0.043 (0.69)	−0.080 (0.45)	0.186 (0.08)	−0.099 (0.35)	0.078 (0.46)	−0.054 (0.61)
	Joint Attention	−0.092 (0.39)	−0.054 (0.61)	0.144 (0.18)	−0.010 (0.93)	0.045 (0.67)	−0.110 (0.30)
	Moving Toy	0.025 (0.82)	0.060 (0.57)	−0.046 (0.67)	0.058 (0.59)	0.019 (0.86)	−0.018 (0.87)
Mental health	Activity	−0.148 (0.16)	0.001 (0.99)	0.085 (0.42)	−0.068 (0.52)	0.075 (0.48)	−0.083 (0.44)
	Dyadic Bid	0.013 (0.90)	−0.057 (0.59)	−0.092 (0.39)	0.032 (0.76)	0.006 (0.95)	−0.101 (0.34)
	Joint Attention	−0.119 (0.26)	−0.049 (0.65)	0.134 (0.21)	0.034 (0.75)	0.056 (0.60)	−0.121 (0.25)
	Moving Toy	−0.078 (0.46)	0.082 (0.44)	−0.011 (0.92)	0.104 (0.33)	−0.084 (0.43)	−0.003 (0.98)
Restrictive repetitive behaviors	Activity	−0.139 (0.19)	0.060 (0.58)	0.134 (0.21)	−0.083 (0.44)	0.056 (0.60)	−0.077 (0.47)
	Dyadic Bid	−0.089 (0.40)	0.018 (0.87)	−0.078 (0.46)	−0.051 (0.63)	0.011 (0.92)	0.003 (0.98)
	Joint Attention	−0.187 (0.08)	0.011 (0.92)	0.024 (0.82)	−0.044 (0.68)	0.145 (0.17)	−0.165 (0.12)
	Moving Toy	−0.154 (0.15)	0.166 (0.12)	−0.023 (0.83)	−0.015 (0.89)	0.058 (0.59)	−0.095 (0.37)
Self-regulation	Activity	−0.023 (0.83)	−0.042 (0.69)	0.056 (0.60)	−0.050 (0.64)	0.083 (0.44)	−0.095 (0.37)
	Dyadic Bid	0.121 (0.25)	−0.151 (0.16)	−0.040 (0.71)	0.123 (0.25)	−0.096 (0.37)	0.058 (0.59)
	Joint Attention	−0.106 (0.32)	−0.139 (0.19)	0.012 (0.91)	0.106 (0.32)	−0.092 (0.39)	0.017 (0.87)
	Moving Toy	−0.018 (0.87)	−0.103 (0.34)	0.121 (0.25)	0.055 (0.60)	0.095 (0.37)	−0.136 (0.20)
Social communication	Activity	**−0.217 (0.04)**	0.147 (0.17)	0.144 (0.17)	−0.042 (0.69)	0.070 (0.51)	−0.084 (0.43)
	Dyadic Bid	−0.116 (0.27)	0.163 (0.12)	0.129 (0.23)	0.063 (0.55)	−0.088 (0.41)	−0.016 (0.88)
	Joint Attention	**−0.237 (0.02)**	**0.245 (0.02)**	0.150 (0.16)	0.048 (0.65)	**0.209 (0.05)**	**−0.343 (10^{-3})**
	Moving Toy	**−0.220 (0.04)**	**0.285 (0.01)**	0.024 (0.83)	0.108 (0.31)	0.036 (0.74)	−0.167 (0.12)
Autism Diagnostic Observation Schedule, 2nd edition (n = 94)							
Restricted and repetitive behavior	Activity	0.101 (0.34)	−0.030 (0.78)	−0.104 (0.32)	−0.103 (0.33)	0.104 (0.33)	0.106 (0.32)
	Dyadic Bid	0.154 (0.14)	0.075 (0.48)	**0.266 (0.01)**	−0.013 (0.91)	−0.065 (0.54)	0.127 (0.23)
	Joint Attention	−0.042 (0.69)	−0.085 (0.42)	0.034 (0.75)	−0.138 (0.19)	0.014 (0.89)	0.109 (0.30)
	Moving Toy	0.098 (0.35)	−0.038 (0.72)	−0.069 (0.52)	−0.009 (0.93)	−0.091 (0.39)	**0.208 (0.05)**
Social affect	Activity	−0.085 (0.43)	0.151 (0.15)	0.057 (0.59)	−0.089 (0.40)	−0.182 (0.08)	0.180 (0.09)
	Dyadic Bid	−0.122 (0.25)	**0.227 (0.03)**	−0.102 (0.34)	−0.145 (0.17)	0.040 (0.71)	0.169 (0.11)
	Joint Attention	−0.039 (0.71)	−0.002 (0.99)	−0.015 (0.89)	−0.174 (0.10)	−0.007 (0.95)	0.136 (0.20)
	Moving Toy	−0.198 (0.06)	0.152 (0.15)	−0.138 (0.19)	−0.128 (0.23)	−0.144 (0.17)	0.156 (0.14)

Continued on the following page

ASD symptom	Region-of-interest<br>Condition	% Valid Time	Background	Body	Hands/Activity	Face	Toys
Total score	Activity	−0.076 (0.47)	0.090 (0.40)	0.006 (0.96)	−0.122 (0.25)	−0.133 (0.21)	0.193 (0.07)
	Dyadic Bid	−0.086 (0.42)	**0.227 (0.03)**	−0.018 (0.87)	−0.173 (0.10)	−0.001 (0.99)	**0.288 (0.01)**
	Joint Attention	−0.081 (0.45)	−0.002 (0.98)	−0.002 (0.98)	**−0.218 (0.04)**	−0.047 (0.66)	0.177 (0.09)
	Moving Toy	−0.141 (0.18)	0.099 (0.35)	−0.167 (0.11)	−0.106 (0.32)	**−0.222 (0.03)**	**0.246 (0.02)**
Aberrant Behavior Checklist (n = 94)							
Hyperactivity non-compliance	Activity	−0.019 (0.86)	−0.090 (0.40)	−0.024 (0.82)	0.027 (0.80)	0.085 (0.42)	−0.137 (0.19)
	Dyadic Bid	0.098 (0.35)	−0.159 (0.13)	0.012 (0.91)	0.156 (0.14)	−0.106 (0.32)	−0.026 (0.80)
	Joint Attention	−0.093 (0.38)	−0.028 (0.79)	0.114 (0.28)	0.047 (0.66)	−0.033 (0.76)	−0.090 (0.40)
	Moving Toy	0.049 (0.65)	0.090 (0.40)	0.097 (0.36)	0.138 (0.19)	0.089 (0.40)	−0.186 (0.08)
Inappropriate speech	Activity	0.039 (0.72)	0.023 (0.83)	−0.025 (0.82)	0.043 (0.68)	0.096 (0.36)	−0.193 (0.07)
	Dyadic Bid	0.072 (0.50)	$4 \cdot 10^{-4}$ (0.996)	−0.013 (0.90)	0.056 (0.60)	−0.030 (0.77)	−0.027 (0.80)
	Joint Attention	−0.097 (0.36)	−0.080 (0.45)	0.038 (0.72)	0.116 (0.27)	0.149 (0.16)	−0.190 (0.07)
	Moving Toy	−0.058 (0.59)	0.091 (0.39)	**0.235 (0.02)**	0.136 (0.20)	0.103 (0.33)	**−0.214 (0.04)**
Irritability	Activity	−0.098 (0.35)	−0.016 (0.88)	0.018 (0.87)	−0.031 (0.77)	0.074 (0.49)	−0.111 (0.30)
	Dyadic Bid	0.067 (0.53)	−0.084 (0.43)	0.018 (0.87)	0.028 (0.79)	0.040 (0.71)	−0.163 (0.12)
	Joint Attention	−0.130 (0.22)	0.006 (0.95)	0.163 (0.12)	−0.017 (0.87)	0.019 (0.86)	−0.126 (0.24)
	Moving Toy	0.058 (0.59)	0.150 (0.16)	0.016 (0.88)	0.094 (0.38)	0.024 (0.82)	−0.070 (0.51)
Lethargy social withdrawal	Activity	−0.029 (0.79)	−0.021 (0.84)	0.005 (0.97)	0.087 (0.41)	−0.016 (0.88)	−0.117 (0.27)
	Dyadic Bid	0.092 (0.38)	−0.015 (0.89)	−0.020 (0.85)	**0.212 (0.04)**	−0.130 (0.22)	−0.002 (0.98)
	Joint Attention	−0.119 (0.26)	0.054 (0.61)	0.154 (0.14)	0.061 (0.57)	0.120 (0.26)	**−0.271 (0.01)**
	Moving Toy	−0.020 (0.85)	**0.213 (0.04)**	0.042 (0.69)	0.162 (0.13)	−0.106 (0.32)	−0.048 (0.65)
Stereotypic behavior	Activity	−0.065 (0.54)	0.069 (0.52)	0.040 (0.70)	0.026 (0.81)	−0.089 (0.40)	0.010 (0.92)
	Dyadic Bid	−0.030 (0.78)	0.107 (0.31)	−0.044 (0.68)	−0.068 (0.52)	0.048 (0.65)	0.004 (0.97)
	Joint Attention	−0.095 (0.37)	−0.021 (0.85)	−0.028 (0.80)	−0.012 (0.91)	0.056 (0.60)	−0.093 (0.38)
	Moving Toy	−0.096 (0.36)	**0.242 (0.02)**	−0.032 (0.77)	0.067 (0.53)	0.059 (0.58)	−0.193 (0.07)
Child Adolescent Symptom Inventory – Anxiety (n = 94)							
Total score	Activity	−0.050 (0.63)	−0.029 (0.79)	0.045 (0.67)	−0.102 (0.33)	0.170 (0.11)	−0.004 (0.97)
	Dyadic Bid	0.074 (0.49)	0.025 (0.81)	−0.100 (0.35)	0.114 (0.28)	−0.071 (0.51)	−0.067 (0.53)
	Joint Attention	−0.037 (0.73)	−0.106 (0.32)	0.112 (0.29)	0.063 (0.55)	0.106 (0.32)	−0.140 (0.18)
	Moving Toy	−0.078 (0.46)	0.160 (0.13)	0.059 (0.58)	0.153 (0.15)	−0.082 (0.44)	−0.015 (0.89)
Repetitive Behavior Scale – Revised (n = 94)							
Compulsive behavior	Activity	0.020 (0.85)	−0.081 (0.44)	−0.030 (0.77)	−0.129 (0.22)	0.087 (0.41)	0.116 (0.27)
	Dyadic Bid	0.061 (0.56)	−0.038 (0.72)	−0.038 (0.72)	−0.097 (0.36)	0.066 (0.53)	0.027 (0.80)
	Joint Attention	0.025 (0.81)	−0.092 (0.39)	0.039 (0.71)	−0.094 (0.37)	0.154 (0.15)	−0.009 (0.93)
	Moving Toy	0.071 (0.51)	0.018 (0.86)	−0.150 (0.16)	−0.134 (0.20)	0.099 (0.35)	0.007 (0.95)

Continued on the following page.

ASD symptom	Region-of-interest<br>Condition	% Valid Time	Background	Body	Hands/Activity	Face	Toys
Ritualistic behavior	Activity	−0.098 (0.36)	−0.005 (0.96)	0.046 (0.67)	−0.072 (0.50)	0.080 (0.45)	−0.028 (0.79)
	Dyadic Bid	−0.079 (0.46)	−0.042 (0.69)	−0.045 (0.67)	−0.023 (0.83)	0.038 (0.72)	−0.059 (0.58)
	Joint Attention	−0.099 (0.35)	−0.130 (0.22)	0.029 (0.78)	−0.078 (0.46)	**0.217 (0.04)**	−0.072 (0.50)
	Moving Toy	−0.141 (0.18)	0.112 (0.29)	−0.040 (0.71)	0.073 (0.49)	0.062 (0.56)	−0.074 (0.48)
Restricted behavior	Activity	−0.047 (0.65)	−0.021 (0.85)	−0.039 (0.71)	−0.106 (0.32)	0.120 (0.26)	0.004 (0.97)
	Dyadic Bid	0.030 (0.78)	0.103 (0.33)	−0.052 (0.62)	−0.167 (0.11)	0.191 (0.07)	−0.036 (0.73)
	Joint Attention	−0.046 (0.66)	0.058 (0.59)	0.043 (0.69)	−0.123 (0.24)	**0.228 (0.03)**	−0.138 (0.19)
	Moving Toy	−0.030 (0.78)	0.193 (0.07)	−0.069 (0.51)	0.002 (0.98)	0.058 (0.58)	−0.057 (0.59)
Sameness behavior	Activity	−0.022 (0.84)	−0.040 (0.71)	0.043 (0.68)	−0.070 (0.51)	0.120 (0.26)	−0.034 (0.75)
	Dyadic Bid	−0.078 (0.46)	0.057 (0.59)	0.028 (0.79)	−0.062 (0.56)	0.102 (0.34)	−0.071 (0.50)
	Joint Attention	−0.118 (0.27)	−0.065 (0.54)	0.100 (0.35)	−0.049 (0.64)	**0.231 (0.03)**	−0.162 (0.13)
	Moving Toy	−0.040 (0.70)	0.135 (0.20)	−0.035 (0.74)	0.070 (0.51)	0.045 (0.67)	−0.060 (0.57)
Self−injurious behavior	Activity	0.065 (0.54)	−0.111 (0.30)	0.068 (0.52)	−0.160 (0.13)	0.084 (0.43)	0.093 (0.38)
	Dyadic Bid	0.037 (0.73)	−0.096 (0.36)	−0.134 (0.20)	−0.085 (0.42)	0.152 (0.15)	−0.056 (0.60)
	Joint Attention	0.029 (0.78)	−0.163 (0.12)	0.054 (0.61)	−0.113 (0.29)	0.192 (0.07)	0.026 (0.81)
	Moving Toy	0.007 (0.94)	−0.119 (0.26)	0.036 (0.74)	−0.071 (0.51)	0.167 (0.11)	−0.064 (0.55)
Stereotyped behavior	Activity	−0.082 (0.44)	0.073 (0.49)	−0.019 (0.86)	0.016 (0.88)	−0.120 (0.26)	0.083 (0.43)
	Dyadic Bid	−0.101 (0.34)	0.128 (0.22)	−0.107 (0.31)	−0.110 (0.30)	0.093 (0.38)	0.081 (0.45)
	Joint Attention	−0.200 (0.06)	0.049 (0.64)	−0.081 (0.45)	0.044 (0.68)	0.063 (0.55)	−0.109 (0.30)
	Moving Toy	−0.078 (0.47)	0.148 (0.16)	−0.102 (0.34)	−0.019 (0.86)	0.092 (0.38)	−0.129 (0.22)
Total score	Activity	−0.084 (0.43)	−0.006 (0.95)	0.028 (0.79)	−0.120 (0.26)	0.107 (0.32)	0.030 (0.78)
	Dyadic Bid	−0.070 (0.51)	0.023 (0.83)	−0.062 (0.56)	−0.098 (0.36)	0.114 (0.28)	−0.027 (0.80)
	Joint Attention	−0.129 (0.22)	−0.066 (0.53)	0.023 (0.83)	−0.085 (0.42)	**0.216 (0.04)**	−0.100 (0.35)
	Moving Toy	−0.067 (0.53)	0.125 (0.24)	−0.093 (0.38)	−0.011 (0.92)	0.091 (0.39)	−0.086 (0.42)
Social Responsiveness Scale 2 (n = 93)							
Social awareness	Activity	0.007 (0.95)	0.015 (0.89)	−0.074 (0.49)	0.084 (0.43)	0.058 (0.58)	−0.129 (0.22)
	Dyadic Bid	−0.004 (0.97)	−0.041 (0.70)	−0.048 (0.65)	0.088 (0.41)	−0.021 (0.84)	−0.084 (0.43)
	Joint Attention	−0.176 (0.10)	0.173 (0.10)	0.074 (0.49)	0.141 (0.19)	0.002 (0.98)	−0.201 (0.06)
	Moving Toy	−0.054 (0.61)	0.087 (0.42)	0.076 (0.48)	0.163 (0.13)	−0.009 (0.94)	−0.113 (0.29)
Social cognition	Activity	0.064 (0.55)	−0.029 (0.79)	−0.113 (0.29)	0.024 (0.82)	0.130 (0.22)	−0.067 (0.53)
	Dyadic Bid	0.104 (0.33)	−0.018 (0.86)	−0.132 (0.21)	0.082 (0.44)	0.006 (0.95)	−0.135 (0.20)
	Joint Attention	−0.124 (0.24)	0.099 (0.35)	−0.029 (0.78)	0.086 (0.42)	0.107 (0.32)	**−0.259 (0.01)**
	Moving Toy	−0.008 (0.94)	0.105 (0.33)	−0.112 (0.29)	0.051 (0.64)	0.033 (0.76)	−0.103 (0.33)

Continued on the following page.

ASD symptom	Region-of-interest<br>Condition	% Valid Time	Background	Body	Hands/Activity	Face	Toys
Social communication	Activity	−0.080 (0.45)	0.005 (0.96)	−0.098 (0.36)	0.062 (0.56)	0.029 (0.79)	−0.126 (0.24)
	Dyadic Bid	0.004 (0.97)	0.030 (0.78)	−0.127 (0.23)	0.044 (0.68)	0.036 (0.74)	−0.109 (0.31)
	Joint Attention	**−0.215 (0.04)**	0.124 (0.24)	0.108 (0.31)	0.119 (0.27)	0.052 (0.63)	**−0.273 (0.01)**
	Moving Toy	−0.112 (0.29)	0.179 (0.09)	0.029 (0.78)	0.170 (0.11)	−0.080 (0.45)	−0.084 (0.43)
Social motivation	Activity	−0.061 (0.57)	0.018 (0.87)	0.043 (0.68)	−0.005 (0.97)	0.131 (0.22)	−0.088 (0.41)
	Dyadic Bid	0.056 (0.60)	0.112 (0.29)	−0.169 (0.11)	0.119 (0.26)	0.010 (0.92)	−0.074 (0.49)
	Joint Attention	−0.058 (0.59)	0.024 (0.82)	0.175 (0.10)	0.084 (0.43)	**0.284 (0.01)**	**−0.352 (10^{-3})**
	Moving Toy	−0.084 (0.43)	0.148 (0.16)	0.005 (0.96)	0.104 (0.33)	0.003 (0.98)	−0.113 (0.29)
Restricted interests and repetitive behavior	Activity	0.015 (0.89)	0.002 (0.99)	0.031 (0.77)	−0.059 (0.58)	0.057 (0.59)	−0.014 (0.90)
	Dyadic Bid	0.108 (0.31)	0.013 (0.90)	−0.110 (0.30)	−0.004 (0.97)	0.033 (0.76)	−0.087 (0.42)
	Joint Attention	−0.134 (0.21)	0.026 (0.81)	0.102 (0.38)	0.020 (0.85)	0.118 (0.27)	**−0.215 (0.04)**
	Moving Toy	−0.042 (0.69)	0.079 (0.46)	0.005 (0.96)	0.049 (0.65)	0.007 (0.95)	−0.071 (0.51)
Social communication and interaction	Activity	−0.046 (0.66)	0.005 (0.96)	−0.065 (0.54)	0.055 (0.61)	0.067 (0.53)	−0.115 (0.28)
	Dyadic Bid	0.026 (0.81)	0.040 (0.71)	−0.153 (0.15)	0.095 (0.37)	0.010 (0.93)	−0.124 (0.24)
	Joint Attention	−0.177 (0.09)	0.117 (0.27)	0.090 (0.40)	0.109 (0.31)	0.107 (0.32)	**−0.295 ($5\cdot10^{-3}$)**
	Moving Toy	−0.091 (0.39)	0.158 (0.14)	0.007 (0.94)	0.142 (0.18)	−0.020 (0.85)	−0.124 (0.24)
Total score	Activity	−0.032 (0.76)	0.005 (0.96)	−0.053 (0.62)	0.015 (0.89)	0.076 (0.47)	−0.082 (0.44)
	Dyadic Bid	0.038 (0.72)	0.030 (0.78)	−0.141 (0.18)	0.057 (0.59)	0.030 (0.78)	−0.116 (0.28)
	Joint Attention	−0.175 (0.10)	0.098 (0.36)	0.086 (0.42)	0.075 (0.48)	0.126 (0.24)	**−0.283 (0.01)**
	Moving Toy	−0.081 (0.45)	0.136 (0.20)	−0.001 (0.99)	0.105 (0.33)	−0.019 (0.86)	−0.099 (0.35)

The data are presented for each behavior rating scale, ASD symptom, stimulus condition and region-of-interest separately. Cells contain Spearman partial correlation coefficients along with the corresponding two-sided P-values in parentheses. The correlation coefficients are computed on the data of all individuals with ASD with participant's age, sex and the KBIT-2 IQ composite score being used as covariates. Cells with P-values below 0.05 are highlighted in bold. 'n' indicates the number of participants. ASD, autism spectrum disorder; IQ, intelligence quotient; KBIT-2, Kaufman Brief Intelligence Test-2.

Supplementary Table 11. Comparison of participants with ASD that were included in analysis versus those excluded due to technical or calibration failures.

Scale	Included in analysis	n	Total score			
			Mean	Standard deviation	Range	Kolmogorov-Smirnov test, *P*-value
Autism Behavior Inventory	Yes	94	1.9	0.8	0.3 – 4.6	0.18
	No	42	2.1	0.8	0.1 – 3.9	
Autism Diagnostic Observation Schedule, 2nd edition	Yes	94	7.5	1.7	4.0 – 10.0	0.91
	No	42	7.8	1.8	4.0 – 10.0	
Child Adolescent Symptom Inventory – Anxiety	Yes	94	17.0	10.0	2.0 – 52.0	0.50
	No	42	16.4	13.2	0.0 – 54.0	
Repetitive Behavior Scale – Revised	Yes	94	27.7	21.9	2.0 – 99.0	0.20
	No	42	29.1	21.8	1.0 – 74.0	
Social Responsiveness Scale 2	Yes	94	74.7	12.0	48.0 – 105.0	0.46
	No	42	74.4	8.8	56.0 – 91.0	
Kaufman Brief Intelligence Test-2	Yes	94	100.3	19.2	60.0 – 136.0	0.21
	No	42	93.5	19.2	60.0 – 132.0	

The two groups of participants are compared in terms of the overall severity of ASD symptoms, as captured by the total score of a given scale. Cells contain mean, standard deviation and range of the total score for each scale and group of participants separately. n indicates the number of participants in each of the two groups. All reported P-values are two-sided

Supplementary Figure 1. Distributions of % *Valid Time* for each stimulus condition and group of participants separately.

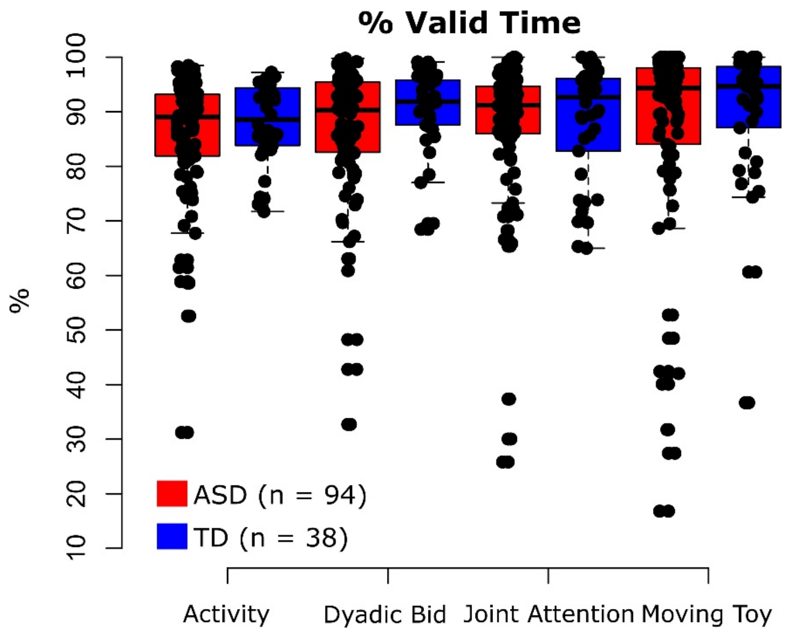

The data are summarized in a form of boxplots. Black dots denote individual participants. The data of individuals with ASD and TD controls are highlighted in red and blue colors, respectively. 'n' indicates the number of participants. Note that there exist no significant between-group differences in % Valid Time in any of the four stimulus conditions (**Supplementary Table 4**). ASD, autism spectrum disorder; TD, typically developing

Supplementary Figure 2. Significant relationships between % *Valid Time*, *% looking time* and the KBIT-2 IQ composite score.

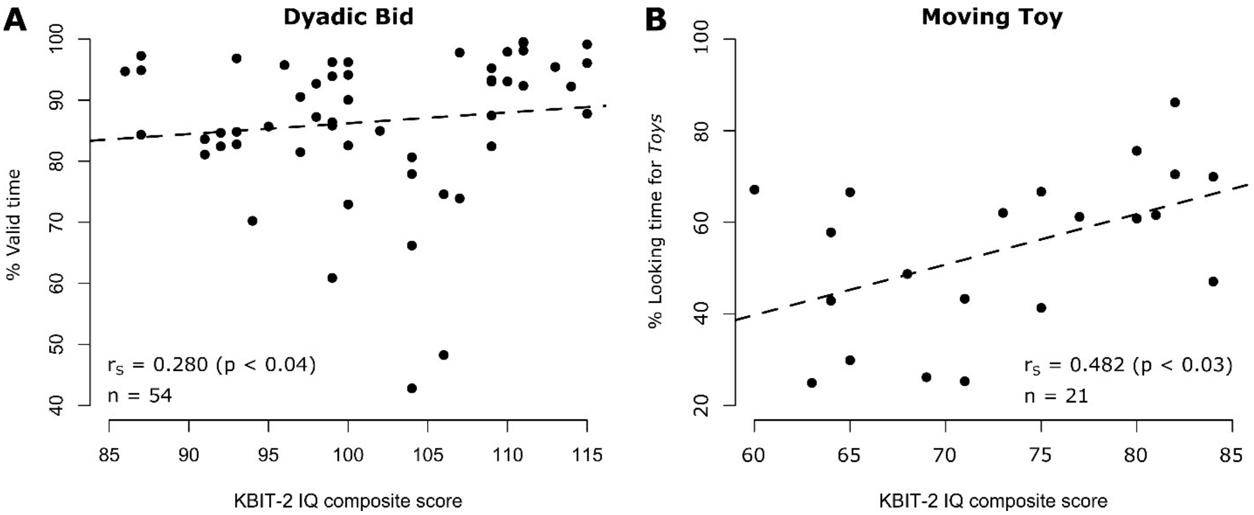

A, Scatter plot between % Valid Time in the condition Dyadic Bid and the KBIT-2 IQ composite score in the group of individuals with ASD with average IQ (range: 85–115). B, Scatter plot between *% looking time* for Toys in the condition Moving Toy and the KBIT-2 IQ composite score in the group of individuals with ASD with low IQ (range: 60–84). Black dots in each panel indicate individual participants. The dashed line in each panel represent the best linear fit of the presented data. r_S and p in each panel correspond to a Spearman correlation coefficient and the corresponding two-sided *P-value* obtained for the presented data (**Supplementary Table 9**). 'n' indicates the number of participants. ASD, autism spectrum disorder; IQ, intelligence quotient; KBIT-2, Kaufman Brief Intelligence Test-
